# Social learning leads to inflexible strategy use in children across three societies

**DOI:** 10.1038/s41598-025-15400-2

**Published:** 2025-08-19

**Authors:** Wilson Vieira, Sarah Pope-Caldwell, Ardain Dzabatou, Luke Maurits, Marie Padberg, Daniel Haun

**Affiliations:** 1 Department of Comparative Cultural Psychology, Max Planck Institute for Evolutionary Anthropology, Deutscher Platz 6, 04103 Leipzig, Germany; 2 Université Marien Ngouabi, Brazzaville, Republic of the Congo

**Keywords:** Cross-cultural psychology, Developmental psychology, Cognitive flexibility, Social learning, Individual learning

## Abstract

Human adaptability relies on our capacity to learn, either from others (socially) or by ourselves (asocially), and to update our strategy repertoire. Cognitive flexibility is the psychological mechanism that mediates strategy use. Humans excel at switching from strategies that no longer work to new ones, but when given the chance to *voluntarily* switch from familiar to better strategies (elective flexibility), we often exhibit remarkable difficulty. However, current research has shown that this may be mediated by exogenous factors such as cultural background and age. The present study used a four-task battery to investigate the impact of social vs. asocial information sources on the development of elective flexibility in children (ages 4 to 14) from three cultural contexts: hunter-gatherer BaYaka; horticulturalists/fisherman Bandongo; and urbanized German. Our data show that German children exhibited overall higher elective flexibility than their Congolese peers whose elective flexibility decreased with age. Interestingly, children across all communities showed less elective flexibility when they learned a strategy socially than when they acquired it themselves. The present study provides important insights on the influence of eco-cultural contexts on the development of adaptive problem-solving, and the impact of social learning on children’s elective flexibility.

**Supplementary Information:**

The online version contains supplementary material available at 10.1038/s41598-025-15400-2.

## Introduction

We think of humans as immensely creative and flexible problem-solvers, and in many ways, this is true. While conducting research in the Republic of the Congo, our local team was using new backpacks to carry research equipment for the upcoming forest expedition. Interestingly, each individual had uniquely changed their backpack using lianas and wood frames in order to make its transportation more comfortable. This is an example, on a microscale, of humans’ ability to flexibly update our strategies and behavior when conditions demand it. Flexible problem-solving is an important skill that promotes adaptability by allowing humans to navigate dynamic environments where we must appropriately apply known strategies or adopt new ones when conditions change. This targeted behavioral adaptation relies on cognitive flexibility: the ability to switch between known, newly acquired, and innovated strategies^1–3^.

There are two scenarios in which humans may need to adjust strategies. The first one is *when* a current strategy stops working, and one *needs* to respond by switching to an alternative. This ability we call *responsive flexibility*^4,5^. Responsive flexibility is important for humans, allowing us to adapt to changing circumstances quickly. The second scenario is when a current strategy continues to work, but a better alternative becomes available. These scenarios, in which one might *voluntarily* switch from a still functional, familiar strategy to an alternative, we call *elective flexibility*^3–8^.

Searching for or trying alternative strategies often incurs time and energy costs, with potentially important consequences for survival and reproduction^9–11^. In responsive flexibility scenarios, moving away from a non-functional strategy clearly outweighs the costs associated with trying or learning another. This explains why humans are proficient in responsive flexibility tasks, even from a young age^12,13^. However, this is not always the case in elective flexibility scenarios.

A classic example of humans’ difficulty to switch away from a functional, familiar strategy is evidenced by Abraham Luchin’s^14^ *Water Jar Task*. In this task, participants learn how to solve a series of arithmetic problems using a time-consuming, three-step strategy, after which they are presented with several new problems that can be solved using either this inefficient but familiar three-step strategy, or a new, one-step strategy. Participants consistently ignored the shortcut across a range of conditions, instead persisting in using their familiar strategy^15–17^. In fact, humans exhibit remarkable difficulty in abandoning familiar strategies for better alternatives, leading to the persistent use of known strategies over unknown, better ones, across a range of cognitive domains^18–20^. This phenomenon, in which familiar strategies ‘block’ better ones, is called *mental* or *cognitive set bias*^8,21,22^.

Considering humans’ incredible proclivity for adaptation and innovation, cognitive set bias seems somewhat paradoxical. In some scenarios, humans seamlessly forsake strategies that do not work, while in others, we seem to use familiar ones obstinately, or at least struggle to use alternatives. To understand when elective flexibility is evoked versus suppressed, we must examine how the costs and benefits of searching for alternatives are contextually mediated.

Social learning mediates elective flexibility by reducing the risks associated with using new strategies. Specifically, social information presents an opportunity to learn valuable strategies that have been “tested” by others, diminishing uncertainty about the outcomes. However, in some scenarios, we may be over-reliant on social information. For example, both children and adults copy irrelevant actions (see^23^ for review) across a range of cultural contexts^24–27^. Indeed, social information can also hinder our ability to find alternative strategies. For example, children who were taught how to interact with a novel object are less explorative than naïve peers^28,29^, and less willing to include an alternative strategy in their repertoire^29^. Thus, even though social information is a valuable tool, it may also boost cognitive set bias.

The present study aims to understand the impact of social learning on elective flexibility in humans and its influence on the development of cognitive set across cultures. Although the development of responsive flexibility has been studied extensively^12,13,30,31^, even cross-culturally^32^, our knowledge regarding the ontogeny of elective flexibility is limited^7,14,33^. Childhood is a fertile period for exploration^34–36^, suggesting that cognitive set might be reduced during this developmental phase. Indeed, Pope and colleagues^6^ found preliminary evidence that American children ages 7–11 were more likely to break cognitive set than adolescents or adults. Additionally, Pope and colleagues^3^ reported reduced susceptibility to cognitive set in Namibian Himba, compared to US American adults. This suggests that cognitive set may be mediated by culture, however, the developmental pathways that lead to cultural variation in cognitive set and elective flexibility are still unclear.

One aspect of our cultural environments that might mediate the development of elective flexibility, particularly when the initially learned information is socially acquired, is the nature of one’s social structure. Traditional hunter-gatherer societies are known for foundational schemas that prioritize individual action-autonomy^37–39^. For instance, in BaYaka culture, one cannot enforce his will on other individuals (including children) and one is not obliged to follow others’ instructions^38^. In this social atmosphere, one might expect individually acquired information to be prioritized more strongly, and conversely, the significance of social learning is less so, when compared to communities that deprioritize individual autonomy^37,40^. Thus, if varying cultural emphasis on individual action autonomy impacts the prioritization of individual vs social learning, social information would be less prioritized in egalitarian societies than in other cultural contexts.

The current study assessed the development of elective flexibility in 157 children from four to 14 years of age, and 28 individual adults tested in at least one of the tasks (here broadly classified as older than 18 years of age), across three cultures: egalitarian, hunter-gatherer BaYaka and hierarchical, fisher-farmer Bandongo from the Republic of the Congo, as well as hierarchical, market-integrated Germans. We focused on the influence of social information on participants’ ability to forsake familiar strategies to adopt a better alternative. We predicted that cognitive set bias would increase with age in all cultures and that socially acquired strategies would be more difficult to switch from (i.e., resulting in higher rates of cognitive set). We also expected that BaYaka participants, who prioritize individual action autonomy, will be less affected by the social condition than the more hierarchical Bandongo and German participants.

To measure cognitive set bias we used four elective flexibility games: Ecological LS-DS task (Eco LS-DS); Lilypad Task (Lilypad); Pin Box Task (Box), and the Maze Task (Maze). These games can be grouped according to their (a) Presentation: Computer games (ECO LS-DS and Maze) and Hands-on games (Box and Lilypad); and (b) Domain: Problem-Solving games (ECO LS-DS and Box) and Navigation games (Lilypad and Maze).

In each game, participants were tested on their ability to abandon a previously acquired learned strategy (LS) for a more efficient, direct strategy (DS), in one of two conditions: social or asocial. Each task was composed of 15 trials divided into five consecutive phases: (1) Acquisition phase: A single trial wherein participants acquired the LS that allows them to solve the task at hand. In this phase, they acquire the LS either by themselves (asocial condition) or by observing the experimenter’s (research assistant of the same ethnicity as the child) demonstration (social condition); (2) Practice phase: Seven trials that can only be solved by using the LS, acquired on the previous phase; (3) Test phase: Four trials that can either be solved using the LS or using the now-available, better alternative, the Direct Strategy (DS) (i.e., shortcut); (4) Extinction phase: One trial that can only be solved by using the DS; and (5) Post-Extinction phase: Two trials that can either be solved by using the familiar LS or the novel DS (i.e., shortcut). For example, during the Box task, participants learn, either by themselves or via a demonstration, how to open a box by removing two pins in sequence and then lifting a clear plastic lid to collect the reward (a ball) inside the box (LS) on trial one. This same sequence (Pin1 → Pin2 → Lid → Reward) is then used to collect the reward for six additional trials. In trials eight and nine, participants are presented with the same box, but this time, the clear plastic lid is replaced with a hollow frame. This allows participants to either collect the reward without removing either the pin or the lid, by simply reaching inside the box (DS) or by continuing to use the familiar Pin1 → Pin2 → Lid → Reward (LS). A similar rationale was used for the Eco LS-DS game. In this task, participants learned that, in order to collect the reward, they needed to select on a touch screen three images in sequence, before finally selecting the reward (LS) after it appeared. During the Test Phase (and Post-Extinction Phase), the reward appeared at the beginning of the trial, allowing participants to either select it immediately (DS) or continue to select Image1 → Image2 → Image3 → Reward(LS). For the Lilypad and the Maze tasks, participants were shown a maze consisting of three trails. In the Lilypad they learned to follow the 8-step yellow trail (LS) to collect the reward, while on the Maze they learned to use the 10 red stepping stones to collect the reward (LS). In both games, the alternative trails did not lead to the reward (i.e., a missing “stepping stone” prevents them from advancing). In the Test and Post-Extinction Phases of both Lilypad and Maze tasks the shorter, 4-step trail (DS) is no longer blocked, allowing participants to use it to obtain the reward. Thus, participants could choose between using the longer trails (LS) and the shorter ones (DS). For a detailed description of each task, see Supplementary Materials, Appendix 3, and the detailed protocols for each task at https://osf.io/4bdfe.

The task battery included both problem-solving and navigation tasks to understand if developmental and cultural variability in cognitive set would be consistent across these cognitive domains, as previous literature has conflated them^14,33^. Tasks were further divided into computer tasks (Eco LS-DS and Maze) and hands-on tasks (Box and Lilypad). We considered it important to include both as the computer tasks allowed us to more directly compare the results of this study to previous developmental assessments of cognitive set^3,14,33^; however, our Congolese communities have limited exposure to digital screens, which might conceivably lead to more conservative responses, especially with children, due to the novelty of screens themselves.

The use of the LS in the presence of the DS, i.e. during the Test phase and the Post-Extinction phase, indicated lower elective flexibility. The use of the DS during the Test phase and Post-Extinction phase indicated higher elective flexibility. Notably, the problem-solving tasks (but not the navigation tasks, offered the possibility of using an intermediate strategy between the LS and the DS, a “Switch-Strategy (SS)” consisting of beginning the LS but switching to the DS part-way through; however, prior to the current study, the SS had been infrequently used in similar tasks^3,7^.

## Results

Age was included as a continuous predictor in the mixed-effects model to retain the full variability across the developmental range (ages 4–14) and to maximize statistical power. This approach avoids the loss of information and potential biases associated with categorizing continuous variables. Although age was modeled as a continuous predictor, the main effect of age represents an average linear trend across the full sample and is conditional on the reference levels of other predictors (e.g., condition, presentation, ethnicity). Given significant interactions involving age—particularly with condition—the age coefficient alone does not fully convey the practical impact of age on strategy use. To interpret and visualize the interactions between age with experimental conditions and predictor variables, we conducted planned contrasts at three representative age points: age three (youngest observed), age nine (sample mean), and age 15 (oldest observed). This approach allows us to illustrate how the influence of experimental conditions varies meaningfully across developmental stages, rather than relying solely on the global age coefficient.

Despite the present study focusing on children from 4 to 14 years old, some of the children were slightly younger or older than the aimed age interval. To make sure that data from all children was used for the analysis and due to the nature of our models that allow us to make accurate predictions (see Analysis section), we decided to run our contrasts and plot model predictions having three years of age as the youngest and 15 as the oldest age group.

When we report below a significant difference between two age groups or conditions, we mean that a 95% HPD interval for the difference in probability of LS use between those groups or conditions excluded zero. Full posterior details can be found in the Supplementary Material.

We aimed to contextualize the child data using a small adult sample. Although this adult data provides a glimpse into the continuity of the age trajectories, we did not formally analyze the adult data due to the low sample size of adults [Box Task: N = 25 (n_BaYaka_ = 8, n_Bandongo_ = 9, n_German_ = 8); Eco LS-DS Task: N = 28 (n_BaYaka_ = 10, n_Bandongo_ = 10, n_German_ = 8); Lilypad Task: N = 15 (n_Bandongo_ = 7 ,n_German_ = 8); Maze Task: N = 20 (n_BaYaka_ = 3, n_Bandongo_ = 10, n_German_ = 7)]. Adult data was plotted by using the proportion of LS and DS answers in each task and condition.

### Test phase

#### Social information condition effects

**Fig. 1:**
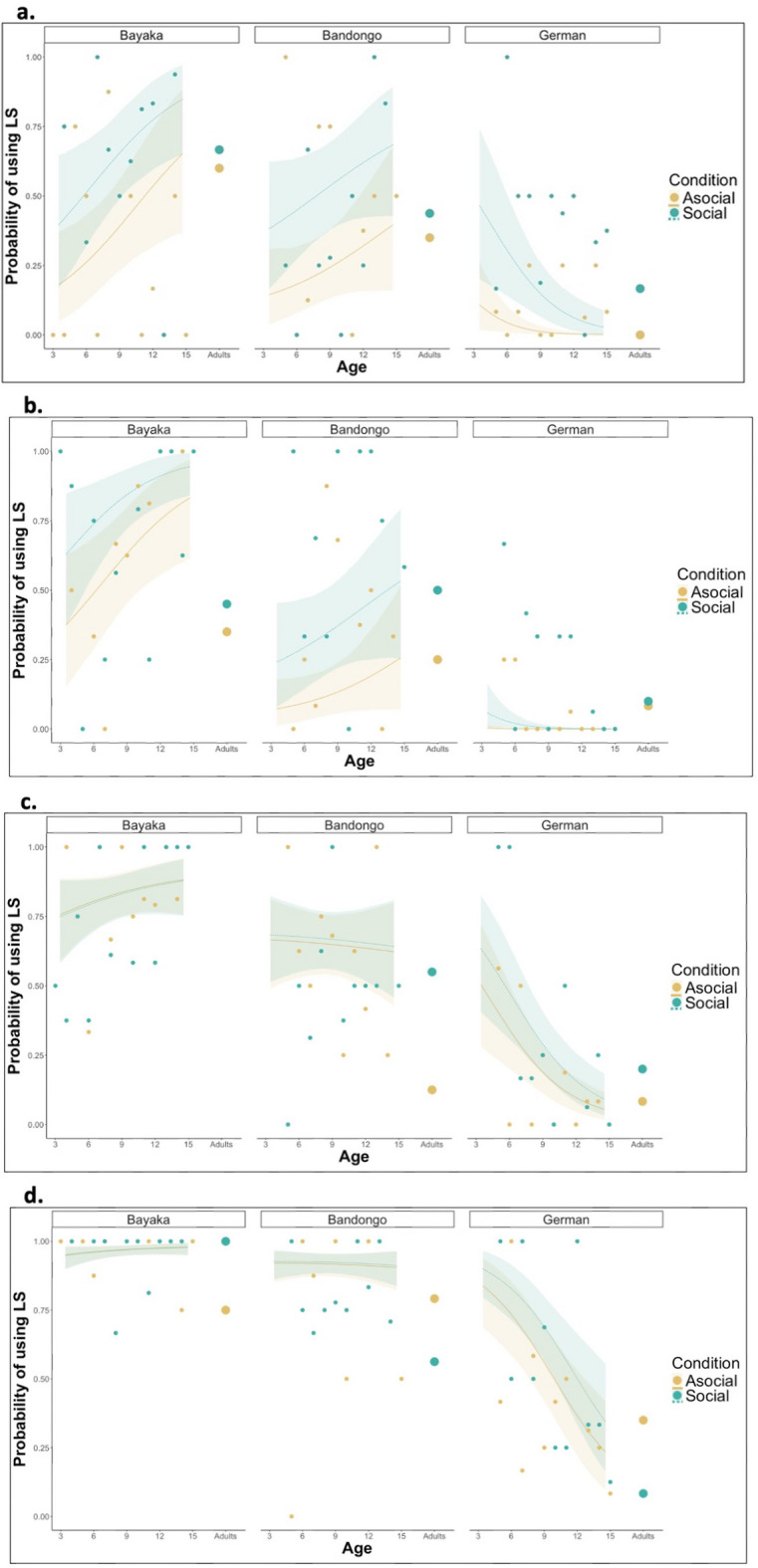
Elective flexibility developmental trajectory—Test Phase: a,b,c,d, show posterior estimates of the probability of using the Learned Strategy (LS) at each given age by children of different cultural backgrounds (egalitarian BaYaka, hierarchical Bandongo, and hierarchical German). Lines show posterior means and ribbons 95% HPD intervals. The trajectory shown is for the middle trial of the Test phase for each of the tasks. Dots represent the mean probability of LS-use (after the four trials) grouped by age and culture in children and adults in both conditions. The Asocial condition is represented by the color yellow ; while the Social condition is represented by the color green. a, Age trajectories for the Pin Box task. N_BaYaka_ = 45; N_Bandongo_ = 47; N_German_ = 52. b, Age trajectories for the Eco LS-DS task. N_BaYaka_ = 50; N_Bandongo_ = 51; N_German_ = 50. c, Age trajectories for the Lilypad task. N_BaYaka_ = 51; N_Bandongo_ = 51; N_German_ = 52. d, Age trajectories for the Maze task, N_BaYaka_ = 47; N_Bandongo_ = 50; N_German_ = 49.

In problem-solving tasks (Box and Eco LS-DS), across all cultural contexts, children were significantly more likely to use the LS in the social condition compared to the asocial condition [Box: 95% HPDI = BaYaka (0.12, 0.37), Bandongo (0.19, 0.40), German (0.08, 0.30); Eco LS-DS 95% HPDI = BaYaka (0.07, 0.29), Bandongo (0.16, 0.37), German (0.00, 0.02)] (Fig. 1, Supplementary Table 1), meaning that they seemed to be less flexible when learned strategies were acquired through demonstration, than through trial-and-error learning. In the navigation tasks (Lilypad and Maze), this effect was found in German children [95% HPDI = Lilypad (0.01, 0.16); Maze (0.02, 0.25)] (Supplementary Table 1) but not in BaYaka and Bandongo children.

#### Age effects

In problem-solving tasks, younger BaYaka children were more flexible, thus less likely to use the LS compared to their older counterparts [Box: 95% HPDI = Asocial (0.11, 0.79), Social (0.09, 0.70); Eco LS-DS 95% HPDI = Asocial (0.10, 0.05); Social (0.05, 0.51)], suggesting that flexibility decreases with age in this culture group. However, for German children, we observed the opposite age effect, such that younger children were more likely to use the LS than older children [Box: 95% HPDI = (Asocial: − 0.34, − 0.02; Social: − 0.79, − 0.17); Eco LS-DS: 95% HPDI = (Asocial: − 0.02, − 0.00; Social: − 0.22, − 0.00) (Fig. 1, Supplementary Table 2), suggesting that flexibility increases with age in this culture group. These effects were found regardless of social information condition for BaYaka and German participants, but no differences in LS-use were found between younger and older Bandongo children in either condition (Supplementary Table 2). In the navigation tasks, we observed that older German participants were significantly more flexible than younger ones, regardless of whether the LS was acquired socially or asocially [Lilypad: 95% HPDI = (Asocial: − 0.62, − 0.17; Social: − 0.72, − 0.24); Maze: 95% HPDI = (Asocial: − 0.81, − 0.36; Social: − 0.80, − 0.34)] (Fig. 1, Supplementary Table 2). Despite our limited adult sample size, it appears that adults’ LS-use is perhaps less affected by the information source of the LS (asocial vs social) and, overall, potentially less likely for Congolese adults. However, overall, adults’ LS-use was mostly in line with the presented childhood trajectories (Fig. 1).

#### Effect of cultural context

Elective flexibility varied across cultures, depending on age. Model predictions based on the youngest children indicate no cross-cultural differences in LS-use on the Pin Box, Maze, or Lilypad tasks; however, on the Eco LS-DS task, young BaYaka children were less flexible, using the LS more often than young Bandongo [95% HPDI = (Asocial: − 0.58, − 0.07; Social: − 0.65, − 0.07)] and young German children [95% HPDI (Asocial: 0.14, 0.67; Social: 0.20, 0.81)] in both conditions and Bandongo children used the LS more often than German children, in the asocial condition only [95% HPDI = (Asocial: 0.00, 0.22)]. (Supplementary Table 3a). At older ages, BaYaka and Bandongo children were significantly more likely to use the LS than German children, and this was found across all tasks, and conditions (Supplementary Table 3b-c), showing higher flexibility from older German children, compared to their Congolese peers. Older BaYaka children were also more likely to use the LS than Bandongo children in the Eco LS-DS, Maze, and Lilypad (but not Pin Box) tasks {[average age: Eco LS-DS 95% HPDI = (Asocial: − 0.70, − 0.33; Social: − 0.60, − 0.26); Lilypad 95% HPDI = (Asocial: − 0.35, − 0.08; Social: − 0.31, − 0.06); Maze tasks 95% HPDI = (Asocial: − 0.12, − 0.02; Social: − 0.11, − 0.02)]; [older age: Eco LS-DS 95% HPDI = (Asocial: − 0.78, − 0.28; Social: − 0.60, − 0.12); Lilypad 95% HPDI = (Asocial: − 0.50, − 0.09; Social: − 0.47, − 0.08); Maze tasks 95% HPDI = (Asocial: − 0.19, − 0.02; Social: − 0.17, − 0.02)]} (Supplementary Table 3b-c).

#### Effect of task presentation and trials

We also examined how task presentation, whether it was conducted on a computer or with physical objects, influenced children’s elective flexibility. In problem-solving tasks (Eco LS-DS and Pin Box), across conditions, BaYaka children were more likely to use the LS (less flexible) in the computer task (Eco LS-DS) [95% HPDI = Asocial (− 0.37, − 0.09); Social (− 0.27, − 0.05)], while Bandongo and German children were less likely to use the LS (more flexible) in the hands-on task [95% HPDI = Bandongo (Asocial: 0.03, 0.23; Social: 0.03, 0.29); German (Asocial: 0.00, 0.04; Social: 0.09, 0.31)] (Pin Box; Fig. 1, Supplementary Table 4a). In the navigation tasks (Maze and Lilypad), all children, in both asocial and social conditions, were more likely to use the LS in the computer task (Maze task; Fig. 1, Supplementary Table 4b), suggesting higher overall flexibility in the physical tasks. Across all ages, tasks, conditions, and cultures, children were significantly more likely to use the LS on the first trial of the Test phase compared to the last trial, indicating that for all participants, flexibility increased with exposure to the DS (Supplementary Table 5a-c).

### Post-extinction phase

#### Social information condition effects

**Fig. 2:**
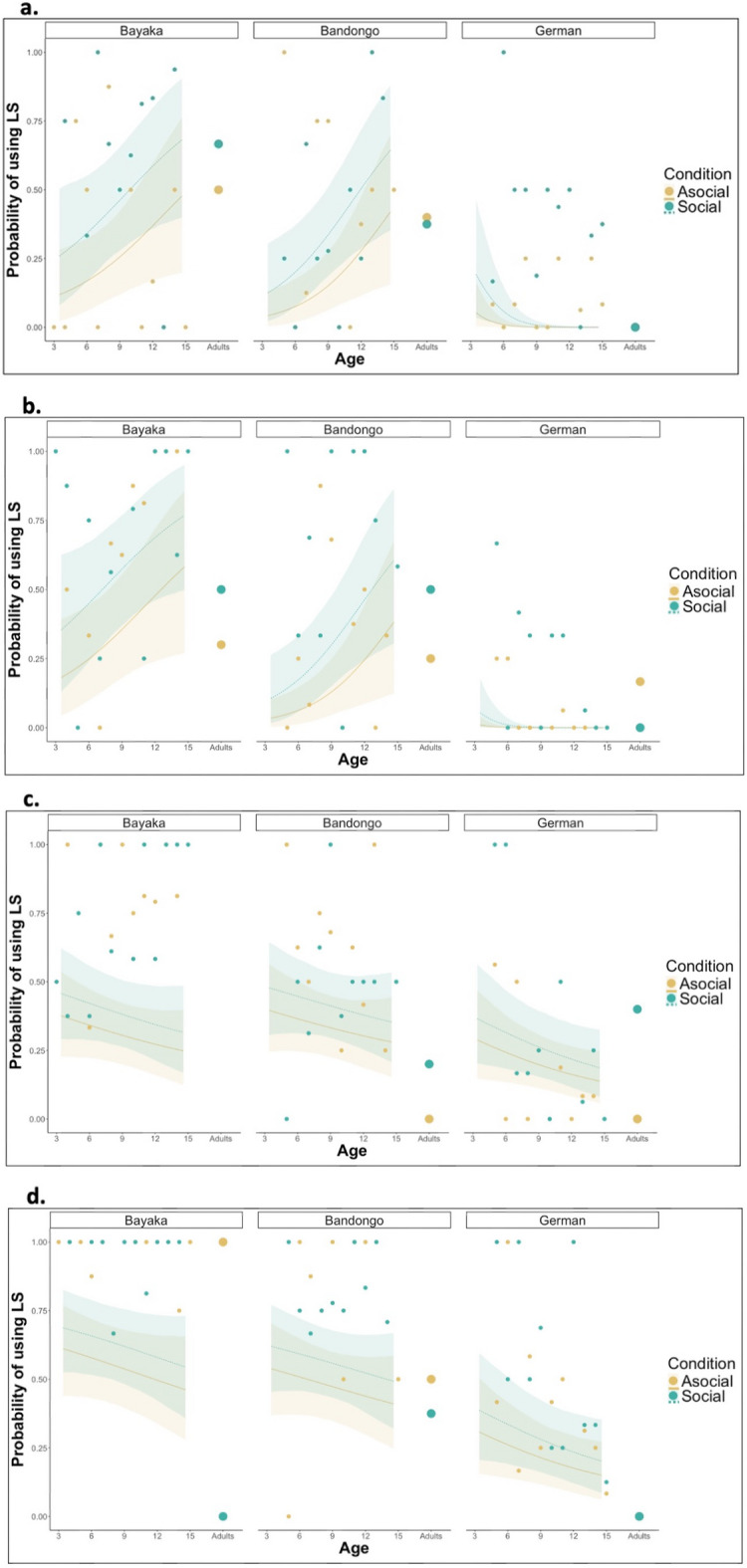
Elective flexibility developmental trajectory—Post-Extinction Phase: a,b,c,d, show posterior estimates of the probability of using the Learned Strategy at each given age by children of different cultural backgrounds (egalitarian BaYaka, hierarchical Bandongo, and hierarchical German). Lines show posterior means and ribbons 95% HPD intervals. The trajectories shown are for the middle trial of the Post-Extinction phase for each of the tasks. Dots represent the mean probability of LS-use (after the two trials) grouped by age and culture in children and adults in both conditions. The Asocial condition is represented by the color yellow; while the Social condition is represented by the color green. a, Age trajectories for the Pin Box task, N_BaYaka_ = 45; N_Bandongo_ = 47; N_German_ = 50. b, Age trajectories for the Eco LS-DS task, N_BaYaka_ = 50; N_Bandongo_ = 51; N_German_ = 50. c, Age trajectories for the Lilypad task, N_BaYaka_ = 51; N_Bandongo_ = 51; N_German_ = 52. d, Age trajectories for the Maze task, N_BaYaka_ = 46; N_Bandongo_ = 50; N_German_ = 49.

Similarly to the Test phase, in problem-solving tasks (Box and Eco LS-DS), across all cultural contexts, children were, significantly less flexible in the social compared to the asocial condition [Box: 95% HPDI = BaYaka (0.06, 0.35), Bandongo (0.09, 0.33), German (0.001, 0.03); Eco LS-DS 95% HPDI = BaYaka (0.06, 0.34), Bandongo (0.08, 0.32), German (0.00, 0.00)] (Fig. 2, Supplementary Table 6), being more likely to use the LS when the strategy was acquired socially. However, in the navigation tasks (Lilypad and Maze), this effect was only found for Bandongo children [95% HPDI = Lilypad (0.00, 0.15); Maze (0.00, 0.16)]; no differences in LS-use between conditions were observed for BaYaka and German children (Fig. 2, Supplementary Table 6).

#### Age effects

The impact of age on LS-use in the Post-Extintion Phase trials mirrors our findings from the Test phase trials. In the problem-solving tasks, younger BaYaka and Bandongo children were less likely to use the LS compared to their older counterparts, who were less flexible [Box: 95% HPDI = BaYaka (Asocial: 0.03, 0.73; Social (0.04, 0.75); Bandongo (Asocial: 0.12, 0.76; Social: 0.18, 0.85); Eco LS-DS: [95% HPDI = BaYaka (Asocial: 0.04, 0.75; Social: 0.04, 0.71); Bandongo (Asocial: 0.10, 0.74; Social: 0.17, 0.84)]; however, for German children, we observed the opposite age effect, such that younger children were more likely to use the LS than older children, showing an increase in flexibility with age [Box: 95% HPDI = (Asocial: − 0.24, − 0.00; Social: − 0.56, − 0.05); Eco LS-DS: 95% HPDI = (Asocial: − 0.06, − 0.00; Social: − 0.25, − 0.00)]. (Fig. 2, Supplementary Table 7). In the navigation tasks, we found no differences between younger and older children’s likelihood of using the LS. Similarly to the Test phase, overall, LS-use in adults was mostly in line with the childhood trajectories, possibly showing little effect of condition (Fig. 2).

#### Effect of cultural context

Cultural context played a similar role in shaping elective flexibility in both the Test phase and Post-Extinction phase. For the problem-solving tasks at younger ages, BaYaka children were less flexible (i.e. used the LS more) than Bandongo (both conditions) [95% HPDI = Asocial: (− 0.43, − 0.01); Social (− 0.59, − 0.01)] and German children (asocial condition) [95% HPDI = Asocial: (0.00, 0.44)] in the Eco LS-DS task (Supplementary Table 8a). While at average ages , German children were more flexible (i.e. less likely to use the LS) compared to Bandongo (except on the Lilypad in the social condition), and BaYaka (except on the Lilypad task) children (Supplementary Table 8b). At this age, BaYaka children are still using LS more often than Bandongo [95% HPDI = Asocial: (− 0.47, − 0.05); Social (− 0.49, − 0.05)] At older ages, no differences in flexibility were found between BaYaka and Bandongo children; however, German children were significantly more flexible (i.e. less likely to use the LS) compared to Bandongo and BaYaka children (except on the Lilypad task in the asocial condition) (Supplementary Table 8c).

#### Effect of task presentation and trials

Unlike the Test phase, task presentation (computer or hands-on) did not influence children’s elective flexibility in problem-solving tasks. However, for navigation tasks, the effect was similar to the Test phase with BaYaka and Bandongo children being less flexible (using the LS more often) in the computer task (Maze) than in the hands-on task (Lilypad) [Maze: 95% HPDI = BaYaka (Asocial: − 0.36, − 0.10; Social (− 0.37, − 0.10); Bandongo (Asocial: − 0.23, − 0.03; Social: − 0.25, − 0.03)] (Supplementary Table 9a,b). Finally, across tasks, ages, conditions, and cultures, there was no effect of trial on the likelihood of using the LS during the Post-Extinction phase, indicating that LS-use did not increase with additional exposure after the extinction trial (Supplementary Table 10a-c).

## Discussion

In the present study, we aimed to study the development of elective flexibility in children from three different cultures and understand the influence that source of information has on cognitive set. Our results provide support for the hypothesis that social learning can suppress flexible problem-solving in humans. Across all cultural contexts, in problem-solving tasks, children who acquired the LS socially were significantly more likely to stick to using the LS rather than switching to a more efficient alternative during the Test phase and, interestingly, these effects carried into the Post Extinction phase, despite children having used the DS during the Extinction trial. Across tasks, we found increased cognitive set in younger compared to older German children, but the opposite was found for BaYaka children, who exhibited increased cognitive set with age. Finally, we found that, overall, Congolese children were more likely to use the LS compared to German children.

One important addition to these findings is that we found somewhat more usage of the SS (switch-strategy) compared to previous research using methods similar to the problem-solving tasks (Eco LS-DS and Box)^3,7^. For example, during the Box task, some participants would start using the LS during the Test phase but then stop, mentioning that the ball was already available through the hollow lid (DS), and then pick it up. Similarly, in other cases, participants mentioned noticing the DS *before* even starting the trial but proceeded to remove one or two pins (part of the LS) before collecting the ball through the hollow lid. To compare elective flexibility across tasks, statistical contrasts were based on the likelihood of using the LS, which is the inverse likelihood of using the DS in navigation tasks, but only one of three strategies (DS, SS, and LS) participants could have used in the problem-solving tasks. Future studies might take advantage of this incremental variation in strategy-use (e.g., shortening the LS by 1 vs 2 steps, or more) by systematically varying the length of available SS strategies.

Social learning is a powerful tool to learn about the world and acquire strategies^41^. However, the current study demonstrates a meaningful *downside* of relying on social information: when children were shown a solution to the problem-solving tasks, their ability to find and adopt a more efficient strategy was suppressed. This finding is in line with previous related research showing that children prioritize information provided by a demonstrator to the extent that they even replicate irrelevant behaviors^23^ or take no notice of non-demonstrated options^28^. However, this is the first study showing that children’s asocial learning (guided by trial-and-error) resulted in more flexible problem-solving when compared with social learning. Additionally, despite reduced overall use of the LS, this effect was also found during the Post-Extinction phase on the problem-solving tasks. Thus, even after successfully using the DS in the extinction trial, participants were still less flexible in the social condition, than in the asocial condition. This suggests that their LS-use stemmed from a belief that the LS was the strategy they *should* use (based on the demonstration of the social partner) (see also^42^). Thus, despite social learning being an advantageous way to quickly and accurately acquire new strategies, it can also lead to detrimental setbacks by preventing users from updating strategies effectively.

Flexibility also varied with age. On the problem-solving tasks, younger Bayaka children were more flexible compared to older children. This developmental trajectory aligns with our previous prediction that flexibility would decrease with age, supported by previous research showing that younger children exhibit increased exploratory behavior which declines with age as they increasingly focus on effective strategies^34,43,44^. Interestingly, the opposite pattern was found for German children, in which older participants were significantly more flexible across tasks than younger children. One explanation for our observed cross-cultural differences in age trajectories relates to the *Variability-Stability-Flexibility* framework, which suggests that behavioral flexibility is achieved at a later stage in development following: first, variability, when successful strategies are sought somewhat randomly; and second, stability, during which successful strategies are honed (Ionescu, 2017). It is possible that the youngest BaYaka participants were exhibiting a variable response style, which later progressed into a more stable reliance on the LS, whereas the youngest German participants may have already been exhibiting a stable reliance on the LS, which then moved toward more flexible strategy use with age.

External eco-cultural factors may contribute to the differences in elective flexibility found between Congolese and German children in this study. At younger ages, relatively fewer differences were found between children across cultures, at older ages, German children were reliably more flexible than their Congolese peers. Adopting an alternative, unknown strategy entails a certain degree of risk; thus, it is possible that different levels of tolerance to risk shaped participants’ use of the known (LS) and unknown (DS) strategies. In fact, Amir and colleagues^45^ found that children living in subsistence-based communities (e.g. farming, hunter-gathering) were less risk-prone when compared to peers living in market-integrated settings. The authors suggest that these differences result from increased food security coming from market integration, which allowed children in these market-integrated societies to make more risky decisions^46–48^. Thus, German children’s increased flexibility compared to Congolese children may have been due to German children’s higher degree of market integration and more predictable environment, which serves as a buffer against the risks of failed strategies. In other words, German children may have been more willing to use the DS because of an increased tolerance to uncertainty, allowing them to shift sooner from the familiar LS to the unknown DS. On the other hand, BaYaka and Bandongo children (who rely on hunting/gathering and fishing/farming, respectively) may not have experienced a level of food security that allows them to forego already tested, functional strategies, over alternatives.

We expected that BaYaka participants, who are part of an egalitarian community, would be less affected by the social condition than the more hierarchical Bandongo and German participants. However, we found no cross-cultural differences in the effect of social condition on LS-use. We interpret this as evidence of a potentially universal impact of social information on flexibility. However, it is also possible that, despite receiving the social information from a Mwaka (sing. BaYaka) experimenter, BaYaka participants were still hesitant to deviate from it based on the presence of the other experimenter(s) from hierarchical communities (e.g. European, Bantu) during testing. Note, BaYaka are typically deferential to the Bandongo/Bantu hierarchical system when in the village, and that may have impacted their responses during the experiments in the presence of culturally non-BaYaka people. Future research might benefit from ensuring cultural homogeneity of all experimenters present in the testing area. Additionally, we characterized participants’ values concerning individual action-autonomy based on their group membership, based on the extensive ethnographic literature^38^, as well as our own experiences and conversations with community members. However, it is possible that intra-group variation in participants’ individual action autonomy could have contributed to the lack of clear group-level effect in our findings. Future research should aim to pair measurements of cognitive flexibility with individual-level measurements of individual action autonomy^49^.

It is important to consider that the cross-cultural differences we found in elective flexibility may be driven by other cultural factors. The main difference between the LS and the DS is that the LS takes more actions to complete (thus, theoretically, more time-consuming). However, it is entirely possible that reducing the number of actions from 4 to 1 (problem-solving tasks); from 8 to 4 (Lilypad task), and 10 to 4 (Maze task), was not tempting enough to encourage the use of the unknown DS. Furthermore, amongst European educational institutions in general, it is expected that children develop some degree of creative thinking^50,51^. German children may have perceived these tasks as pedagogical games in which they should be creative and “think outside the box.”. For example, on several occasions, German children stated to the experimenter how “smart” and “attentive” they were by having spotted the alternative strategy when it was available. Contrary to their German peers, Congolese children may have been more motivated by the reward and less concerned with the time it took to get it.

Due to the broad age range of participants in this study, we aimed to create tasks that were accessible to children of all ages. It is possible that compared to the previous elective flexibility studies that used arithmetic problems^14^ and geometric shapes^3,6^, the tasks used in this study may have been easier for German children in general, allowing them to locate alternative strategies with ease. In the studies from Pope and colleagues^3,6^, participants were presented with BASE trials (where they could only use LS) or PROBE trials (where they could use either LS or DS) in a random order. During these trials, the sequence was shown briefly, requiring participants to keep strategies in their working memory. This meant that the task used in those studies may have been more taxing for participants, since they had to keep the LS in their working memory at all times, leading to a lower probability of using DS when available. In fact, in another study using the same task, but in which working memory was reduced, researchers found lower rates of LS use^8^. However, it is important to note that all participants, including Germans, used the LS more on the first trial of the Test phase, compared to the last trial (Supplementary Fig. 1–2; Supplementary Table 5a-c). Thus, even German children overcame an initial tendency toward cognitive set over the course of the four consecutive Test phase trials, and even perhaps generalized this information across tasks (given the similar design).

The current study used a four-task battery to assess the extent to which cognitive set bias extends across cognitive domains (navigation vs. problem-solving, hereafter problem types) and presentation type (i.e., hands-on vs. computer, hereafter presentation styles). It was especially important to us to include hands-on tasks in addition to computer tasks, given that BaYaka and Bandongo children have limited to no contact with digital technology. We found that both presentation style and problem type influenced LS use during the Test phase. Specifically, before the extinction trial on the problem-solving tasks, BaYaka children used the LS more in the computer (Eco LS-DS) compared to the hands-on task (Pin Box), while German and Bandongo children showed the opposite inclination. In the navigation tasks, all children used the LS more in the computer task (Maze) compared to the hands-on task (Lilypad). It is possible that this effect was driven by cross-cultural differences in screen familiarity. For example, during the Test phase of the Eco LS-DS, children only needed to touch the image of a ball on the screen to use the DS strategy, making it a simple and obvious cue. However, BaYaka children, who have slightly less experience with screens than Bandongo, and much less than German children, might have perceived the computer task’s DS as less salient.

Similarly, although we did not statistically test whether domain impacted LS use (see Methods), a cursory look suggests that Congolese children’s LS use may have been higher in the navigation compared to problem-solving tasks. One explanation for this could be that the navigation tasks required participants to adhere to a set of arbitrary rules (e.g., if the trail is blocked, follow it back and start again), which made these tasks more demanding to master. On several occasions, we observed that Congolese children (particularly BaYaka) would *fail* to follow the rules given by the instructor (e.g., following a direct line to the reward instead of following the trail presented; ignoring the *barrier* in the trail and trying to reach the reward) and had to be continuously instructed about it, sometimes for several experimental trials, before mastering the basic game rules*.* These types of arbitrary rules are common in board and video game scenarios, making them much more familiar for Germans compared to BaYaka and Bandongo participants. Thus, the fact that Congolese children used the LS more in navigation tasks may have been caused by them placing a higher value on the LS due to the added effort associated with learning it. Future studies are needed to directly assess the influence of task difficulty on cognitive set. However, these results provide preliminary support for the idea that flexibility is suppressed by increasing task complexity^16^ (1950, pp. 279–297).

Elective flexibility is a multi-faceted construct. The current study took an important first step toward identifying how elective flexibility is influenced by social learning throughout development. Our findings suggest that, even though social learning is an important tool for learning about our world, it can also suppress elective flexibility. When we consider that building upon or replacing functional strategies is at the core of our species’ cumulative cultural evolution, identifying the factors influencing elective flexibility is critical to understanding the origins of our uniquely human cultural diversity.

## Methods

The present study was pre-registered on Open Science Framework. All information about the hypothesis, sample size, procedure, incorporated variables, and planned analysis can be found at https://osf.io/4bdfe. All deviations from the pre-registration are explicitly detailed below. All data and analyses are available on the repository associated with the pre-registration.

### Participants

Data were collected from 157 children (BaYaka: N = 51, N_Female_ = 22, Mean_age_ = 9.23, SD_age_ = 3.15; Bandongo: N = 54, N_Female_ = 32, Mean_age_ = 9.49, SD_age_ = 2.95; German: N = 52, N_Female_ = 25, Mean_age_ = 9.84, SD_age_ = 3.28) and 28 adults (BaYaka: N = 10, N_Female_ = 6, Mean_age_ = 35.8, SD_age_ = 12.01; Bandongo: N = 10, N_Female_ = 4, Mean_age_ = 25.7, SD_age_ = 9.80; German: N = 8, N_Female_ = 3, Mean_age_ = 35.2, SD_age_ = 9.58). Congolese participants were recruited from a small village (~ 800 inhabitants) in the Likouala Department of the Republic of the Congo between September and October 2021 for children, and between August and September 2022 for adults. Testing took place in a research house and a large testing tent in forest camps. Age estimates (informed by local research assistants and all available information) were used when the exact age of the children was unknown. German participants were tested in the ChildLab at the Max Planck Institute for Evolutionary Anthropology in Leipzig between January and June 2022 for children, and between June and October 2022 for adults. Parental informed consent and child assent were obtained for all child participants, and informed consent was obtained for all adult participants. All study procedures were approved by the Ethics Council of the Max Planck Society and followed the Ethical guidelines detailed in Bruno et al.^52^.

### Ethnographic context

The BaYaka are one of several nomadic hunter-gatherer groups living in the Congo River basin^53^. BaYaka mainly rely on foraging for forest resources (e.g., honey, wild yam, mushrooms, wild game, leaves, fruits, seeds, and fish); however, their diet is occasionally supplemented by agricultural products (e.g., plantain, cassava) derived from small-scale, low-intensity agricultural practices^37,54^. The BaYaka are further characterized by the absence of food storage methods, a mark of their immediate-return economy^55^. Like many other forager groups, the BaYaka are characterized by an egalitarian social organization^38^, in which there are few age hierarchies and sex-based differences and an absence of formal leaders^39^, as well as respect for individual autonomy (Boyette et al., 2020) and sharing of resources and parental care^38^.

The Bandongo are one of the Bantu-speaking cultural groups also occupying the Congo River basin. Bandongo mainly rely on fishing and shifting agriculture as means of subsistence, as well as hunting, often with the aid of their BaYaka neighbors^56^. Bandongo culture is characterized by hierarchical social organization with marked age and sex differences in labor, authority, and wealth accumulation^40^.

Both BaYaka and Bandongo communities have limited exposure to market goods (e.g., machetes, sandals, fishing hooks). Due to their nomadic lifestyle, formal education is limited amongst BaYaka children, while Bandongo children more often attend school in the village or nearby towns.

German participants were recruited in the city of Leipzig (~ 600,000 inhabitants) located in Saxony, Germany. Germany rates 5th place in the list of highest GDP countries^57^. Likely, participants in this study belong to mid-to-high socio-economic backgrounds. Children in this context are exposed to capitalist, market-integrated contexts, meaning that to obtain food resources, it is necessary to exchange money for food items in markets, shops, etc. Like other European societies, these participants live in a liberal democracy that values individual political and civil freedom^58^. However, it is also characterized by health stratification based on one’s socio-economic status and hierarchies and power asymmetries defined by one’s position within society (Toelstede, 2020). According to UNESCO’s Education Index^59^, Germany rates as the highest index in the world. Formal education is mandatory for children starting at around six years of age.

### Task battery

To measure cognitive set bias, we used a task battery composed of two problem-solving tasks (Box and Eco LS-DS tasks) and two navigation tasks (Lilypad and Maze tasks), in which participants were tested on their ability to abandon a previously learned strategy (LS) for a more efficient, direct strategy (DS). In each game, participants learned (socially or asocially) how to use one strategy to collect a ball, and then they were presented with a better alternative.

We divided each task into five phases:

1. Acquisition phase in which participants learned to solve the task using a somewhat tedious, learned strategy (LS). They acquired this LS either from a same-ethnicity experimenter (social condition) or by themselves through trial and error (asocial condition).
2. Practice phase, consisting of seven trials only solvable using the LS.
3. Test phase, four trials solvable both by the LS and a more efficient strategy, the direct strategy (DS). These trials are the first time that participants can use a shortcut.
4. Extinction phase, a single trial that could only be solved using the DS shortcut, and
5. Post-Extinction phase, two trials which, again, could be solved using either the LS or the DS shortcut (for more details, see Methods section).

Experimenters were not aware of the hypotheses regarding age or condition (Asocial or Social). The experiment was conducted in a within-subject study design, meaning that each participant was assigned to each of the four tasks in a different condition (social or asocial). Thus, each participant was meant to complete two tasks in the social and two asocial conditions. Conditions were counterbalanced across participants. Care was taken in order not to present two consecutive tasks of the same Presentation or Domain, resulting in four possible combinations of task order and task condition. The assignment of combinations to participants was pseudo-randomized, meaning that, initially, combinations of tasks were assigned randomly, but as the study progressed, the number of potential participants decreased, and combinations were assigned to balance the number of participants per gender or to replace dropouts. For a detailed description of each task, see the Supplementary Materials, Appendix 4, and the detailed protocols for each task at https://osf.io/4bdfe.

### Analysis

The methods of analysis used in this project were adapted and modified from the originally pre-registered ones (see https://osf.io/4bdfe for details). Due to the unexpected use of the SS in problem-solving tasks, which was not available in the navigation tasks, these were analyzed separately. For the Test and Post-Extinction Phases, to analyze problem-solving tasks, we used a series of Bayesian ordinal outcome regression models (M1) to predict the likelihood of the use of three strategies: LS, SS, and DS, which the model treated as lying along an ordinal scale, e.g. with SS indicating more flexibility than LS and DS more than SS (LS < SS < DS); while for Navigation Tasks, we used a series of Bayesian binomial regression models (M2) to predict the likelihood of the choosing one of the two strategies: LS and DS. All models used the *brms* (Bürker, 2017, 2018) package in R (R Core Team, 2022).

We stipulated that LS use would be predicted by: i) the age of the participant, with younger children being more likely to use the LS than older ones; ii) their ethnicity (BaYaka, Bandongo, and German), iii) how participants learned the LS strategy (condition social or condition asocial), with children who learned the LS socially being more likely to use it than their peers who learned it asocially, and iv) the presentation of the task (computer or hands-on tasks). Additionally, we expected the trial number in the Test phase and Post-Extinction phase, and the task number (position in which each task is presented to the participant) would influence children’s tendency to use the LS.

For each task, a series of models were fit to predict strategy-use. In all models, random intercepts of both participant ID and ethnicity were included. In those models including interactions between ethnicity and other predictors, these were implemented as random slopes of the other predictors, grouped by ethnicity. A regularizing exponential prior was used for random effect variances, and this encourages pooling data across ethnicities to allow better estimation of common effects in the absence of strong evidence for cross-cultural variation.

Our baseline models (M1.0 and M2.0) included effects of trial and task number. In Models M1.1 and M2.1, we added an effect of age including an interaction with ethnicity. In Model M1.2 and M2.2, we also included an effect of condition including an interaction with ethnicity. In Models M1.3 and M2.3, we included an effect of presentation including an interaction with ethnicity. Finally, in Models M1.4 and M2.4, we added interactions between condition, age and ethnicity (implemented as per-ethnicity random slopes of the condition by age interaction terms). The models used for analysis LS use during the Post-Extinction phase had the same structure as the models used during the Test phase, although they were given different names: M3.0, M3.1, M3.2, M3.3 and M3.4 for Problem-Solving tasks; and M4.0, M4.1, M4.2, M4.3 and M4.4 for Navigation tasks.

To determine the best-fitting of these models, we compared the models’ widely applicable information criterion (WAIC)^60^. We considered one model to outperform another when the difference in WAIC scores exceeded two standard errors. When there was not a unique best performing model by this criteria we considered the simplest of those models whose performance could not be distinguished. The SI tables and model information can be found on Supplementary materials, Appendix 2 (M1.3, 2.3, 3.3 and 4.3). The posterior predictions of the model selected by this procedure were used to calculate the relevant contrasts and plots showing the impact of age, condition, ethnicity, trial, and presentation on the likelihood of using LS.

## Supplementary Information

Below is the link to the electronic supplementary material.

Supplementary Material 1

## Data Availability

De-identified data, protocols, and R scripts used for analysis are publicly available at Open Science Framework (https://osf.io/4bdfe).
