# Supplementary material for: Social learning leads to inflexible strategy use in children across three societies
Source: Sci Rep. 2025 Aug 19;15:30281. doi: 10.1038/s41598-025-15400-2 (PMC12361463; doi:10.1038/s41598-025-15400-2)

**Supplementary Material**

### Appendix 1



**Supplementary Fig. 1: LS use across trials - children data:** The image shows the percentage of  LS-use in each trial of the Test Phase (TS) (trials 8-11) (Pin Box task: N_BaYaka_ = 45; N_Bandongo_ = 47; N_German_ = 52; Eco LS-DS task: N_BaYaka_ = 50; N_Bandongo_ = 51; N_German_ = 50; Lilypad task: N_BaYaka_ = 51; N_Bandongo_ = 51; N_German_ = 52; Maze: N_BaYaka_ = 47; N_Bandongo_ = 50; N_German_ = 49) and Post-Extinction phase (trials 13 and 14)(Pin Box task: N_BaYaka_ = 45; N_Bandongo_ = 47; N_German_ = 50; Eco LS-DS task: N_BaYaka_ = 50; N_Bandongo_ = 51; N_German_ = 50; Lilypad task: N_BaYaka_ = 51; N_Bandongo_ = 51; N_German_ = 52; Maze task: N_BaYaka_ = 46; N_Bandongo_ = 50; N_German_ = 49) by children of the three cultural groups.



**Supplementary Fig. 2: LS use across trials - adult data:** The image shows the percentage of  LS-use in each trial of the Test Phase (TS) (trials 8-11) (Pin Box task: N_BaYaka_ = 8; N_Bandongo_ = 9; N_German_ = 8; Eco LS-DS task: N_BaYaka_ = 10; N_Bandongo_ = 10; N_German_ = 8; Lilypad task: N_BaYaka_ = 0; N_Bandongo_ = 7; N_German_ = 8; Maze: N_BaYaka_ = 3; N_Bandongo_ = 10; N_German_ = 8) and Post-Extinction phase (trials 13 and 14)(Pin Box task: N_BaYaka_ = 8; N_Bandongo_ = 9; N_German_ = 8; Eco LS-DS task: N_BaYaka_ = 10; N_Bandongo_ = 10; N_German_ = 8; Lilypad task: N_BaYaka_ = 0; N_Bandongo_ = 7; N_German_ = 8; Maze task: N_BaYaka_ = 3; N_Bandongo_ = 10; N_German_ = 8) by adults of the three cultural groups.

### Appendix 2

**Test phase Contrasts**

**Model specification**

family: cumulative

Links: mu = logit; disc = identity

FORMULA
*strat_use ~ 1 + condition + presentation + age + trial + task_number + (1 | pid) + (1 + condition + presentation + age | ethnicity)*

Number of observations (Box & LS-DS): 1180

Number of observations (Lilypad & Maze): 1200
Draws: 4 chains, each with iter = 2000; warmup = 1000; thin = 1; total post-warmup draws = 4000

**Model M1.3** (Box & LS-DS):

**
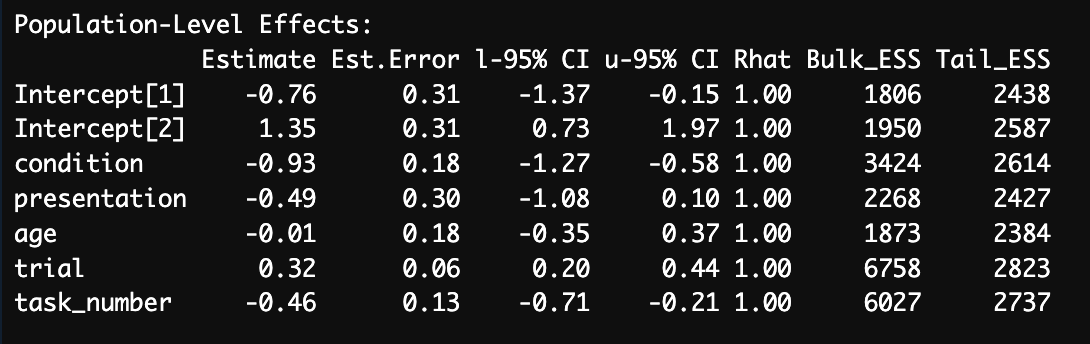
**

**Model M2.3** (Lilypad & Maze):


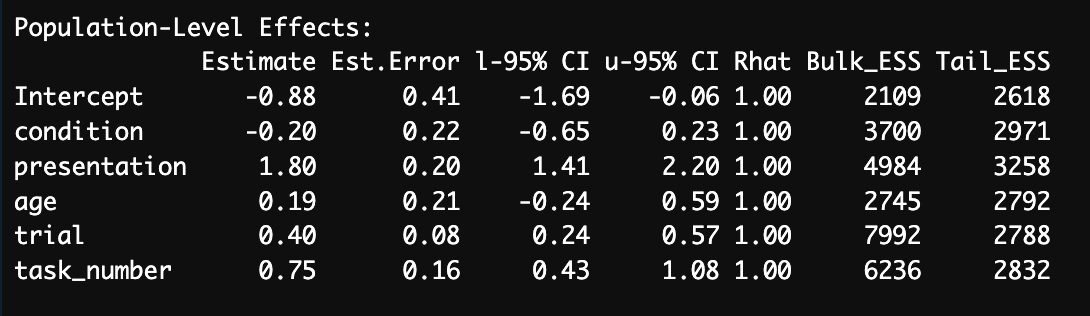


**Model contrasts**

**Supplementary Table 1. Social Information condition effects:** Condition contrasts for the likelihood of using the LS during the Test phase, between the social and asocial condition. The results show that when BaYaka and Bandongo participants acquired the Learned Strategy (LS) socially, they were less likely to use the alternative Direct Strategy on the Box and Eco LS-DS tasks [Box: 95% HPDI = BaYaka (0.12, 0.37), Bandongo (0.19, 0.40); Eco LS-DS 95% HPDI = BaYaka (0.07, 0.29), Bandongo (0.16, 0.37)]. However, this effect was not found for the Lilypad and Maze tasks. The influence of social information on the use of the LS was observed in all tasks in German children [95% HPDI = Box (0.08, 0.30); Eco LS-DS (0.00, 0.02); Lilypad (0.01, 0.16); Maze (0.02, 0.25)].

|  | **Box** | | **Eco LS-DS** | | **Lilypad** | | **Maze** | |
| --- | --- | --- | --- | --- | --- | --- | --- | --- |
|  | 5% | 95% | 5% | 95% | 5% | 95% | 5% | 95% |
| BaYaka | **0.12** | **0.37** | **0.07** | **0.29** | -0.09 | 0.06 | -0.02 | 0.01 |
| Bandongo | **0.19** | **0.40** | **0.16** | **0.37** | -0.07 | 0.10 | -0.03 | 0.04 |
| German | **0.08** | **0.30** | **0.00** | **0.02** | **0.01** | **0.16** | **0.02** | **0.25** |

**Supplementary Table 2. Age effects:** Age contrasts for the likelihood of using the Learned Strategy (LS) during the Test Phase, between the oldest and the youngest children. The results show that older BaYaka children were more likely to use the LS, in both conditions of the Box task [95% HPDI = Asocial (0.11, 0.79); Social (0.09, 0.70)]; and the Eco LS-DS [95% HPDI = Asocial (0.10, 0.05); Social (0.05, 0.51)] task than younger ones. The opposite effect of age was observed on the German children across all tasks, with older children being less likely to use the LS compared to younger ones in both conditions [95% HPDI = Box (Asocial: -0.34, -0.02; Social: -0.79, -0.17); Eco LS-DS (Asocial: -0.02, -0.00; Social: -0.22, -0.00); Lilypad (Asocial: -0.62, -0.17; Social: -0.72, -0.24); Maze (Asocial:-0.81, -0.36; Social: -0.80, -0.34). No effect was found for Bandongo children.

|  | | **Box** | | **Eco LS-DS** | | **Lilypad** | | **Maze** | |
| --- | --- | --- | --- | --- | --- | --- | --- | --- | --- |
|  |  | 5% | 95% | 5% | 95% | 5% | 95% | 5% | 95% |
| BaYaka | Asocial | **0.11** | **0.79** | **0.10** | **0.72** | -0.09 | 0.40 | -0.02 | 0.11 |
|  | Social | **0.09** | **0.70** | **0.05** | **0.51** | -0.09 | 0.41 | -0.02 | 0.12 |
| Bandongo | Asocial | -0.05 | 0.62 | -0.04 | 0.53 | -0.32 | 0.26 | -0.15 | 0.10 |
|  | Social | -0.06 | 0.64 | -0.06 | 0.66 | -0.32 | 0.25 | -0.14 | 0.10 |
| German | Asocial | **-0.34** | **-0.02** | **-0.02** | **-0.00** | **-0.62** | **-0.17** | **-0.81** | **-0.36** |
|  | Social | **-0.79** | **-0.17** | **-0.22** | **-0.00** | **-0.72** | **-0.24** | **-0.80** | **-0.34** |

**Supplementary Table 3. Effect of cultural context:** Cultural background contrasts for the likelihood of using the LS during the Test Phase at: a) youngest (3 y.o); b)  middle (9 y.o) and; c) oldest (15 y.o) ages, between Bandongo and BaYaka, Bandongo and German, and BaYaka and German. The results show that, at (a) at younger ages, in both conditions, BaYaka children are more likely to use the LS than their Bandongo [95% HPDI = (Asocial: -0.58, -0.07; Social: -0.65, -0.07)] and German peers [95% HPDI (Asocial: 0.14, 0.67; Social: 0.20, 0.81)] on the Eco LS-DS task, while Bandongo are more likely to use LS on the Asocial condition of the Eco LS-DS task than German [95% HPDI = (Asocial: 0.00, 0.22)]. At (b) average ages, German children are overall more likely to use the DS instead of the LS across tasks and conditions, compared to Bandongo and BaYaka children, while BaYaka are more likely to use the LS in the Eco LS-DS [95% HPDI = (Asocial: -0.70, -0.33; Social: -0.60, -0.26)], Lilypad [95% HPDI = (Asocial: -0.35, -0.08; Social: -0.31, -0.06)] and Maze tasks [95% HPDI = (Asocial: -0.12, -0.02; Social: -0.11, -0.02)], than Bandongo children in both conditions. The same influence of culture is found at older ages.

a.  Youngest Age

| **Bandongo and BaYaka** | **Box** | | **Eco LS-DS** | | **Lilypad** | | **Maze** | |
| --- | --- | --- | --- | --- | --- | --- | --- | --- |
|  | 5% | 95% | 5% | 95% | 5% | 95% | 5% | 95% |
| Asocial | -0.25 | 0.18 | **-0.58** | **-0.07** | -0.28 | 0.14 | -0.11 | 0.04 |
| Social | -0.32 | 0.31 | **-0.65** | **-0.07** | -0.26 | 0.17 | -0.10 | 0.06 |

| **Bandongo and German** | **Box** | | **Eco LS-DS** | | **Lilypad** | | **Maze** | |
| --- | --- | --- | --- | --- | --- | --- | --- | --- |
|  | 5% | 95% | 5% | 95% | 5% | 95% | 5% | 95% |
| Asocial | -0.30 | 0.24 | **0.00** | **0.22** | -0.19 | 0.38 | -0.07 | 0.24 |
| Social | -0.55 | 0.23 | -0.09 | 0.44 | -0.29 | 0.27 | -0.11 | 0.13 |

| **BaYaka and German** | **Box** | | **Eco LS-DS** | | **Lilypad** | | **Maze** | |
| --- | --- | --- | --- | --- | --- | --- | --- | --- |
|  | 5% | 95% | 5% | 95% | 5% | 95% | 5% | 95% |
| Asocial | -0.30 | 0.31 | **0.14** | **0.67** | -0.16 | 0.50 | -0.05 | 0.28 |
| Social | -0.57 | 0.26 | **0.20** | **0.81** | -0.29 | 0.36 | -0.09 | 0.17 |

b.  Average Age

| **Bandongo and BaYaka** | **Box** | | **Eco LS-DS** | | **Lilypad** | | **Maze** | |
| --- | --- | --- | --- | --- | --- | --- | --- | --- |
|  | 5% | 95% | 5% | 95% | 5% | 95% | 5% | 95% |
| Asocial | -0.36 | 0.04 | **-0.70** | **-0.33** | **-0.35** | **-0.08** | **-0.12** | **-0.02** |
| Social | -0.28 | 0.07 | **-0.60** | **-0.26** | **-0.31** | **-0.06** | **-0.11** | **-0.02** |

| **Bandongo and German** | **Box** | | **Eco LS-DS** | | **Lilypad** | | **Maze** | |
| --- | --- | --- | --- | --- | --- | --- | --- | --- |
|  | 5% | 95% | 5% | 95% | 5% | 95% | 5% | 95% |
| Asocial | **0.16** | **0.44** | **0.08** | **0.30** | **0.29** | **0.55** | **0.28** | **0.55** |
| Social | **0.23** | **0.59** | **0.29** | **0.60** | **0.22** | **0.49** | **0.16** | **0.42** |

| **BaYaka and German** | **Box** | | **Eco LS-DS** | | **Lilypad** | | **Maze** | |
| --- | --- | --- | --- | --- | --- | --- | --- | --- |
|  | 5% | 95% | 5% | 95% | 5% | 95% | 5% | 95% |
| Asocial | **0.28** | **0.63** | **0.54** | **0.86** | **0.51** | **0.75** | **0.35** | **0.62** |
| Social | **0.31** | **0.70** | **0.78** | **0.95** | **0.41** | **0.67** | **0.22** | **0.48** |

c.  Oldest Age

| **Bandongo and BaYaka** | **Box** | | **Eco LS-DS** | | **Lilypad** | | **Maze** | |
| --- | --- | --- | --- | --- | --- | --- | --- | --- |
|  | 5% | 95% | 5% | 95% | 5% | 95% | 5% | 95% |
| Asocial | -0.52 | 0.07 | **-0.78** | **-0.28** | **-0.50** | **-0.09** | **-0.19** | **-0.02** |
| Social | -0.34 | 0.06 | **-0.60** | **-0.12** | **-0.47** | **-0.08** | **-0.17** | **-0.02** |

| **Bandongo and German** | **Box** | | **Eco LS-DS** | | **Lilypad** | | **Maze** | |
| --- | --- | --- | --- | --- | --- | --- | --- | --- |
|  | 5% | 95% | 5% | 95% | 5% | 95% | 5% | 95% |
| Asocial | **0.22** | **0.71** | **0.11** | **0.56** | **0.31** | **0.69** | **0.51** | **0.82** |
| Social | **0.46** | **0.88** | **0.34** | **0.83** | **0.30** | **0.67** | **0.40** | **0.76** |

| **BaYaka and German** | **Box** | | **Eco LS-DS** | | **Lilypad** | | **Maze** | |
| --- | --- | --- | --- | --- | --- | --- | --- | --- |
|  | 5% | 95% | 5% | 95% | 5% | 95% | 5% | 95% |
| Asocial | **0.44** | **0.91** | **0.68** | **0.97** | **0.64** | **0.91** | **0.63** | **0.90** |
| Social | **0.64** | **0.95** | **0.88** | **0.99** | **0.59** | **0.89** | **0.50** | **0.84** |

**Supplementary Table 4. Effect of task presentation:** Presentation contrasts for the likelihood of using the LS during the Test phase, between a) the Pin Box and Eco LS-DS tasks, and b) Lilypad and Maze tasks. The results show that, in both conditions of the problem-solving tasks (a), BaYaka children were less likely to use the LS on the Box Task, compared to the Eco LS-DS [95% HPDI = Asocial (-0.37, -0.09); Social (-0.27, -0.05)]; while Bandongo and German children where more likely to use the LS on the Eco LS-DS task [95% HPDI = Bandongo (Asocial: 0.03, 0.23; Social: 0.03, 0.29); German (Asocial: 0.00, 0.04; Social: 0.09, 0.31)]. In the navigation tasks (b), results show that across conditions, children from all cultures were more likely to use the LS on the Maze task.

a.  Problem-Solving Tasks

| **Box vs Eco LS-DS** | | **5%** | **95%** |
| --- | --- | --- | --- |
| BaYaka | Asocial | **-0.37** | **-0.09** |
|  | Social | **-0.27** | **-0.05** |
| Bandongo | Asocial | **0.03** | **0.23** |
|  | Social | **0.03** | **0.29** |
| German | Asocial | **0.00** | **0.04** |
|  | Social | **0.09** | **0.31** |

b.  Navigation Tasks

| **Lilypad vs Maze** | | **5%** | **95%** |
| --- | --- | --- | --- |
| BaYaka | Asocial | **-0.25** | **-0.10** |
|  | Social | **-0.26** | **-0.11** |
| Bandongo | Asocial | **-0.40** | **-0.24** |
|  | Social | **-0.26** | **-0.11** |
| German | Asocial | **-0.41** | **-0.23** |
|  | Social | **-0.46** | **-0.29** |

**Supplementary Table 5. Effect of task trial:** Trial contrasts for the likelihood of using the LS at: a) youngest (3 y.o); b)  middle (9 y.o) and; c) oldest (15 y.o) ages, between the last trial (trial 11) and the first trial (trial 8) of the Test phase. Across ages, tasks, and conditions, children from all cultures were more likely to use the Direct Strategy instead of the LS.

| Trial 11- Trial 8 | | **Box** | | **Eco LS-DS** | | **Lilypad** | | **Maze** | |
| --- | --- | --- | --- | --- | --- | --- | --- | --- | --- |
|  |  | 5% | 95% | 5% | 95% | 5% | 95% | 5% | 95% |
| BaYaka | Asocial | **-0.26** | **-0.06** | **-0.30** | **-0.12** | **-0.23** | **-0.09** | **-0.11** | **-0.02** |
|  | Social | **-0.30** | **-0.13** | **-0.29** | **-0.10** | **-0.23** | **-0.09** | **-0.11** | **-0.02** |
| Bandongo | Asocial | **-0.25** | **-0.05** | **-0.19** | **-0.02** | **-0.24** | **-0.11** | **-0.13** | **-0.03** |
|  | Social | **-0.30** | **-0.13** | **-0.29** | **-0.08** | **-0.24** | **-0.11** | **-0.13** | **-0.03** |
| German | Asocial | **-0.27** | **-0.04** | **-0.05** | **-0.00** | **-0.24** | **-0.11** | **-0.19** | **-0.04** |
|  | Social | **-0.29** | **-0.11** | **-0.23** | **-0.02** | **-0.24** | **-0.09** | **-0.14** | **-0.02** |

1. Average age

| Trial 11- Trial 8 | | **Box** | | **Eco LS-DS** | | **Lilypad** | | **Maze** | |
| --- | --- | --- | --- | --- | --- | --- | --- | --- | --- |
|  |  | 5% | 95% | 5% | 95% | 5% | 95% | 5% | 95% |
| BaYaka | Asocial | **-0.31** | **-0.15** | **-0.28** | **-0.12** | **-0.19** | **-0.07** | **-0.05** | **-0.01** |
|  | Social | **-0.28** | **-0.11** | **-0.19** | **-0.05** | **-0.19** | **-0.08** | **-0.05** | **-0.01** |
| Bandongo | Asocial | **-0.29** | **-0.13** | **-0.24** | **-0.08** | **-0.25** | **-0.12** | **-0.12** | **-0.04** |
|  | Social | **-0.30** | **-0.15** | **-0.31** | **-0.15** | **-0.25** | **-0.12** | **-0.11** | **-0.04** |
| German | Asocial | **-0.06** | **-0.00** | **-0.00** | **-0.00** | **-0.14** | **-0.05** | **-0.25** | **-0.12** |
|  | Social | **-0.25** | **-0.09** | **-0.03** | **-0.00** | **-0.19** | **-0.07** | **-0.24** | **-0.12** |

1. Oldest age

| Trial 11 vs Trial 8 | | **Box** | | **LS-DS** | | **Lilypad** | | **Maze** | |
| --- | --- | --- | --- | --- | --- | --- | --- | --- | --- |
|  |  | 5% | 95% | 5% | 95% | 5% | 95% | 5% | 95% |
| BaYaka | Asocial | **-0.28** | **-0.08** | **-0.22** | **-0.03** | **-0.17** | **-0.04** | **-0.05** | **-0.00** |
|  | Social | **-0.21** | **-0.02** | **-0.12** | **-0.00** | **-0.18** | **-0.04** | **-0.05** | **-0.00** |
| Bandongo | Asocial | **-0.30** | **-0.14** | **-0.29** | **-0.10** | **-0.25** | **-0.11** | **-0.15** | **-0.03** |
|  | Social | **0.28** | **-0.08** | **-0.30** | **-0.12** | **-0.24** | **-0.11** | **-0.14** | **-0.03** |
| German | Asocial | **-0.01** | **-0.00** | **-0.00** | **-0.00** | **-0.06** | **-0.01** | **-0.19** | **-0.05** |
|  | Social | **-0.14** | **-0.01** | **-0.00** | **-0.00** | **-0.09** | **-0.01** | **-0.22** | **-0.08** |

**Post-Extinction phase**

**Model specification**

family: cumulative

Links: mu = logit; disc = identity

FORMULA
*strat_use ~ 1 + condition + presentation + age + trial + task_number + (1 | pid) + (1 + condition + presentation + age | ethnicity)*

Number of observations (Box & LS-DS): 692

Number of observations (Lilypad & Maze): 183
Draws: 4 chains, each with iter = 2000; warmup = 1000; thin = 1; total post-warmup draws = 4000

**Model M3.3** (Box & LS-DS):

**
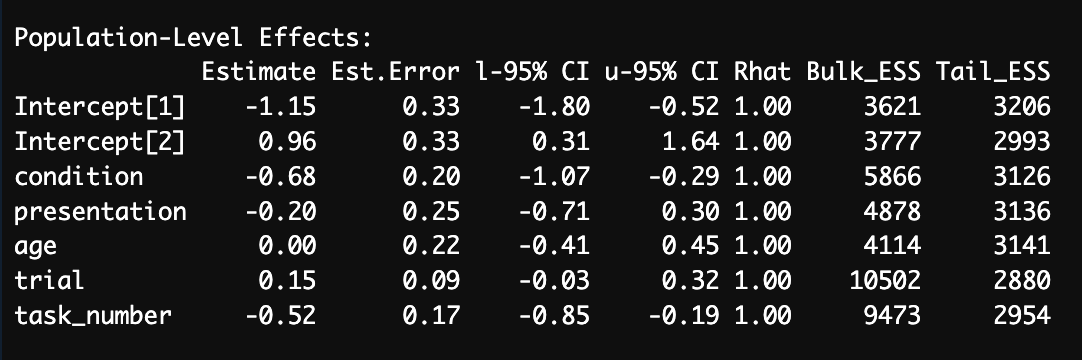
**

**Model M4.3** (Lilypad & Maze):

**
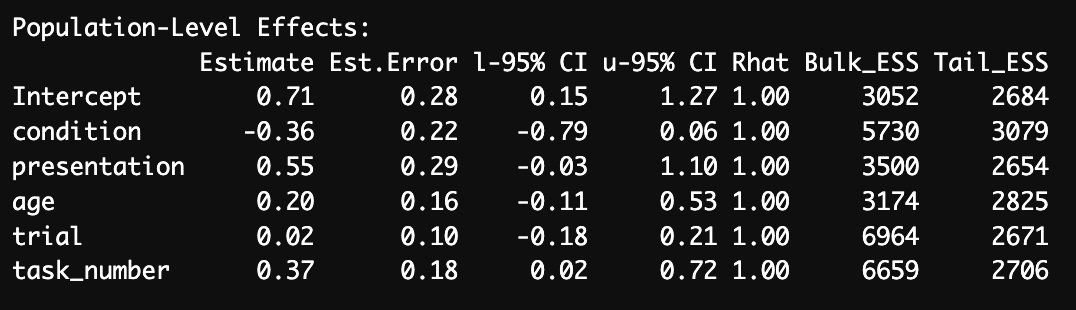
**

**Model contrasts**

**Supplementary Table 6. Social information condition effects:** Condition contrasts for the likelihood of using the LS during the Post-Extinction phase, between the social and asocial condition. The results show that when BaYaka, Bandongo and German participants acquired the Learned Strategy (LS) socially, they were less likely to use the alternative Direct Strategy on the Box and Eco LS-DS tasks [Box: 95% HPDI = BaYaka (0.06, 0.35), Bandongo (0.09, 0.33), German (0.001, 0.03); Eco LS-DS 95% HPDI = BaYaka (0.06, 0.34), Bandongo (0.08, 0.32), German (0.00, 0.00)]. However, this effect was also found for the Lilypad and Maze tasks in Bandongo children [95% HPDI = Lilypad (0.00, 0.15); Maze (0.00, 0.16).

| **Social - Asocial** | **Box** | | **LSDS** | | **Lilypad** | | **Maze** | |
| --- | --- | --- | --- | --- | --- | --- | --- | --- |
|  | 5% | 95% | 5% | 95% | 5% | 95% | 5% | 95% |
| BaYaka | **0.06** | **0.35** | **0.06** | **0.34** | -0.00 | 0.14 | -0.00 | 0.17 |
| Bandongo | **0.09** | **0.33** | **0.08** | **0.32** | **0.00** | **0.15** | **0.00** | **0.16** |
| German | **0.001** | **0.03** | **0.00** | **0.00** | -0.00 | 0.12 | -0.00 | 0.13 |

**Supplementary Table 7.  Age effects:** Age contrasts for the likelihood of using the LS during the Post-Extinction Phase, between the oldest and the youngest children. The results show that older BaYaka and Bandongo children were more likely to use the LS, in both conditions of the Box task [95% HPDI = BaYaka (Asocial: 0.03, 0.73; Social (0.04, 0.75); Bandongo (Asocial: 0.12, 0.76; Social: 0.18, 0.85)], and Eco LS-DS Task [95% HPDI = BaYaka (Asocial: 0.04, 0.75; Social: 0.04, 0.71); Bandongo (Asocial: 0.10, 0.74; Social: 0.17, 0.84)]. The opposite effect of age was observed on the German children across all tasks, with older children being less likely to use the LS compared to younger ones in both conditions [95% HPDI = Box (Asocial: -0.24, -0.00; Social: -0.56, -0.05); Eco LS-DS (Asocial: -0.06, -0.00; Social: -0.25, -0.00)].

| **Oldest - Youngest** | | **Box** | | **Eco LS-DS** | | **Lilypad** | | **Maze** | |
| --- | --- | --- | --- | --- | --- | --- | --- | --- | --- |
|  |  | 5% | 95% | 5% | 95% | 5% | 95% | 5% | 95% |
| BaYaka | Asocial | **0.03** | **0.73** | **0.04** | **0.75** | -0.29 | 0.06 | -0.36 | 0.08 |
|  | Social | **0.04** | **0.75** | **0.04** | **0.71** | -0.33 | 0.07 | -0.36 | 0.07 |
| Bandongo | Asocial | **0.12** | **0.76** | **0.10** | **0.74** | -0.30 | 0.10 | -0.35 | 0.11 |
|  | Social | **0.18** | **0.85** | **0.17** | **0.84** | -0.33 | 0.11 | -0.35 | 0.12 |
| German | Asocial | **-0.24** | **-0.00** | **-0.06** | **-0.00** | -0.32 | 0.00 | -0.34 | 0.00 |
|  | Social | **-0.56** | **-0.05** | **-0.25** | **-0.00** | -0.38 | 0.00 | 0.00 | 0.00 |

**Supplementary Table 8. Effect of cultural context:** Cultural background contrasts for the likelihood of using the LS during the Post-Extinction Phase at: a) youngest (3 y.o); b)  middle (9 y.o) and; c) oldest (15 y.o) ages, between Bandongo and BaYaka, Bandongo and German, and BaYaka and German. The results show that, at (a.) younger ages, BaYaka children are more likely to use the LS than Bandongo children in both conditions of the Eco LS-DS task [95% HPDI = Asocial: (-0.43, -0.01); Social (-0.59, -0.01)], and than German children in the asocial condition of the same task [95% HPDI = Asocial: (0.00, 0.44)]. At (b.) average age, BaYaka children are less likely to use the LS than their Bandongo peers in the Eco LS-DS task in both conditions [95% HPDI = Asocial: (-0.47, 0.05); Social (-0.49, 0.05)], while German children are less likely to use the LS than their Bandongo peers (except on the Lilypad task on the Social condition) and less likely than their BaYaka peers (except on the Lilypad task in both conditions. At (c.) older ages, German children are more likely to use the DS than their Bandongo peers across conditions and tasks, and more likely than BaYaka children to use the alternative strategy (with the exception of the Lilipad task on the asocial condition).

a.  Youngest Age

| **Bandongo - BaYaka** | **Box** | | **Eco LS-DS** | | **Lilypad** | | **Maze** | |
| --- | --- | --- | --- | --- | --- | --- | --- | --- |
|  | 5% | 95% | 5% | 95% | 5% | 95% | 5% | 95% |
| Asocial | -0.29 | 0.04 | **-0.43** | **-0.01** | -0.12 | 0.16 | -0.25 | 0.08 |
| Social | -0.43 | 0.09 | **-0.59** | **-0.01** | -0.13 | 0.17 | -0.23 | 0.08 |

| **Bandongo - German** | **Box** | | **Eco LS-DS** | | **Lilypad** | | **Maze** | |
| --- | --- | --- | --- | --- | --- | --- | --- | --- |
|  | 5% | 95% | 5% | 95% | 5% | 95% | 5% | 95% |
| Asocial | -0.32 | 0.08 | -0.09 | 0.11 | -0.12 | 0.28 | -0.02 | 0.42 |
| Social | -0.59 | 0.12 | -0.28 | 0.21 | -0.14 | 0.30 | -0.03 | 0.43 |

| **BaYaka - German** | **Box** | | **Eco LS-DS** | | **Lilypad** | | **Maze** | |
| --- | --- | --- | --- | --- | --- | --- | --- | --- |
|  | 5% | 95% | 5% | 95% | 5% | 95% | 5% | 95% |
| Asocial | -0.25 | 0.27 | **0.00** | **0.44** | -0.14 | 0.27 | **0.04** | **0.50** |
| Social | -0.48 | 0.34 | -0.08 | 0.60 | -0.16 | 0.29 | **0.04** | **0.52** |

b.  Average Age

| **Bandongo - BaYaka** | **Box** | | **Eco LS-DS** | | **Lilypad** | | **Maze** | |
| --- | --- | --- | --- | --- | --- | --- | --- | --- |
|  | 5% | 95% | 5% | 95% | 5% | 95% | 5% | 95% |
| Asocial | -0.30 | 0.05 | **-0.47** | **-0.05** | -0.08 | 0.13 | -0.22 | 0.08 |
| Social | -0.33 | 0.08 | **-0.49** | **-0.05** | -0.09 | 0.15 | -0.21 | 0.08 |

| **Bandongo - German** | **Box** | | **Eco LS-DS** | | **Lilypad** | | **Maze** | |
| --- | --- | --- | --- | --- | --- | --- | --- | --- |
|  | 5% | 95% | 5% | 95% | 5% | 95% | 5% | 95% |
| Asocial | **0.10** | **0.35** | **0.08** | **0.32** | **0.00** | **0.24** | **0.08** | **0.39** |
| Social | **0.26** | **0.58** | **0.22** | **0.56** | -0.00 | 0.28 | **0.09** | **0.42** |

| **BaYaka - German** | **Box** | | **Eco LS-DS** | | **Lilypad** | | **Maze** | |
| --- | --- | --- | --- | --- | --- | --- | --- | --- |
|  | 5% | 95% | 5% | 95% | 5% | 95% | 5% | 95% |
| Asocial | **0.18** | **0.52** | **0.26** | **0.64** | -0.03 | 0.23 | **0.13** | **0.48** |
| Social | **0.36** | **0.72** | **0.48** | **0.83** | -0.04 | 0.26 | **0.13** | **0.51** |

c.  Oldest Age

| **Bandongo - BaYaka** | **Box** | | **Eco LS-DS** | | **Lilypad** | | **Maze** | |
| --- | --- | --- | --- | --- | --- | --- | --- | --- |
|  | 5% | 95% | 5% | 95% | 5% | 95% | 5% | 95% |
| Asocial | -0.40 | 0.30 | -0.55 | 0.15 | -0.08 | 0.15 | -0.23 | 0.12 |
| Social | -0.32 | 0.26 | -0.45 | 0.12 | -0.10 | 0.18 | -0.23 | 0.12 |

| **Bandongo - German** | **Box** | | **Eco LS-DS** | | **Lilypad** | | **Maze** | |
| --- | --- | --- | --- | --- | --- | --- | --- | --- |
|  | 5% | 95% | 5% | 95% | 5% | 95% | 5% | 95% |
| Asocial | **0.23** | **0.76** | **0.18** | **0.74** | **0.00** | **0.27** | **0.08** | **0.42** |
| Social | **0.46** | **0.91** | **0.40** | **0.90** | **0.00** | **0.32** | **0.09** | **0.46** |

| **BaYaka - German** | **Box** | | **Eco LS-DS** | | **Lilypad** | | **Maze** | |
| --- | --- | --- | --- | --- | --- | --- | --- | --- |
|  | 5% | 95% | 5% | 95% | 5% | 95% | 5% | 95% |
| Asocial | **0.27** | **0.82** | **0.38** | **0.89** | -0.02 | 0.24 | **0.11** | **0.48** |
| Social | **0.49** | **0.92** | **0.61** | **0.96** | **0.24** | **0.28** | **0.12** | **0.54** |

**Supplementary Table 9.  Effect of task presentation:** Presentation contrasts for the likelihood of using the LS during the Post-Extinction Phase, between a) the Pin Box and Eco LS-DS tasks, and b) Lilypad and Maze tasks. The results do not show any effect of presentation for (a) problem-solving tasks. For (b) navigation tasks, BaYaka and Bandongo children across conditions were more likely to use the LS on the Maze task [95% HPDI = BaYaka (Asocial: -0.36, -0.10; Social (-0.37, -0.10); Bandongo (Asocial: -0.23, -0.03; Social: -0.25, -0.03)].

a.  Problem-Solving Tasks

| **Box - Eco LS-DS** |  | **5%** | **95%** |
| --- | --- | --- | --- |
| BaYaka | Asocial | -0.29 | 0.06 |
|  | Social | -0.28 | 0.06 |
| Bandongo | Asocial | -0.07 | 0.14 |
|  | Social | -0.10 | 0.19 |
| German | Asocial | 0.00 | 0.00 |
|  | Social | 0.00 | 0.03 |

b.  Navigation Tasks

| **Lilypad - Maze** |  | **5%** | **95%** |
| --- | --- | --- | --- |
| BaYaka | Asocial | **-0.36** | **-0.10** |
|  | Social | **-0.37** | **-0.10** |
| Bandongo | Asocial | **-0.23** | **-0.03** |
|  | Social | **-0.25** | **-0.03** |
| German | Asocial | -0.10 | 0.08 |
|  | Social | -0.13 | 0.10 |

**Supplementary Table 10. Effect of task trial:** Trial contrasts for the likelihood of using the LS at: a) youngest (3 y.o); b)  middle (9 y.o) and; c) oldest (15 y.o) ages, between the last trial (trial 14) and the first trial (trial 13) of the Post-Extinction phase. The results show no effect of trial across age, cultures, conditions, and tasks.

1. Youngest age

| Trial 14 - Trial 13 | | **Box** | | **Eco LS-DS** | | **Lilypad** | | **Maze** | |
| --- | --- | --- | --- | --- | --- | --- | --- | --- | --- |
|  |  | 5% | 95% | 5% | 95% | 5% | 95% | 5% | 95% |
| BaYaka | Asocial | -0.15 | -0.00 | -0.17 | -0.00 | -0.07 | 0.05 | -0.08 | 0.06 |
|  | Social | -0.19 | -0.00 | -0.20 | -0.00 | -0.08 | 0.06 | -0.07 | 0.06 |
| Bandongo | Asocial | -0.09 | -0.00 | -0.07 | -0.00 | -0.07 | 0.06 | -0.08 | 0.06 |
|  | Social | -0.15 | -0.00 | -0.14 | -0.00 | -0.08 | 0.06 | -0.07 | 0.06 |
| German | Asocial | -0.14 | -0.00 | -0.06 | -0.00 | -0.06 | 0.05 | -0.07 | 0.05 |
|  | Social | -0.19 | -0.00 | -0.14 | -0.00 | -0.07 | 0.05 | -0.07 | 0.06 |

1. Average age

| Trial 14 - Trial 13 | | **Box** | | **Eco LS-DS** | | **Lilypad** | | **Maze** | |
| --- | --- | --- | --- | --- | --- | --- | --- | --- | --- |
|  |  | 5% | 95% | 5% | 95% | 5% | 95% | 5% | 95% |
| BaYaka | Asocial | -0.20 | -0.00 | -0.21 | -0.00 | -0.06 | 0.05 | -0.08 | 0.06 |
|  | Social | -0.21 | -0.00 | -0.20 | -0.00 | -0.07 | 0.06 | -0.08 | 0.06 |
| Bandongo | Asocial | -0.17 | -0.00 | -0.15 | -0.00 | -0.07 | 0.05 | -0.08 | 0.06 |
|  | Social | -0.21 | -0.00 | -0.21 | -0.00 | -0.07 | 0.06 | -0.08 | 0.06 |
| German | Asocial | -0.00 | -0.00 | -0.00 | -0.00 | -0.05 | 0.03 | -0.05 | 0.04 |
|  | Social | -0.02 | -0.00 | -0.00 | -0.00 | -0.06 | 0.04 | -0.06 | 0.05 |

1. Oldest age

| Trial 14 - Trial 13 | | **Box** | | **Eco LS-DS** | | **Lilypad** | | **Maze** | |
| --- | --- | --- | --- | --- | --- | --- | --- | --- | --- |
|  |  | 5% | 95% | 5% | 95% | 5% | 95% | 5% | 95% |
| BaYaka | Asocial | -0.20 | -0.00 | -0.19 | -0.00 | -0.05 | 0.04 | -0.08 | 0.06 |
|  | Social | -0.18 | -0.00 | -0.16 | -0.00 | -0.06 | 0.05 | -0.08 | 0.06 |
| Bandongo | Asocial | -0.20 | -0.00 | -0.20 | -0.00 | -0.06 | 0.05 | -0.07 | 0.06 |
|  | Social | -0.18 | -0.00 | -0.19 | -0.00 | -0.07 | 0.05 | -0.08 | 0.06 |
| German | Asocial | -0.00 | -0.00 | -0.00 | -0.00 | -0.03 | 0.02 | -0.04 | 0.03 |
|  | Social | -0.00 | -0.00 | -0.00 | -0.00 | -0.04 | 0.03 | -0.05 | 0.04 |

### Appendix 3

**Supplementary Fig.3: Elective flexibility developmental trajectories - Test Phase: a,b,c,d,** show posterior estimates of the probabilities of using a given strategy at each given age by children of different cultural backgrounds (egalitarian BaYaka, hierarchical Bandongo, and hierarchical German). Lines show posterior means and ribbons 95% HPD intervals.  The trajectory shown is for the middle trial of the Test phase for each of the tasks. Red lines and triangles represent Learned Strategy (LS), grey lines and triangles represent Switch Strategy (SS) (only present for Pin Box and Eco LS-DS tasks) and blue lines and triangles represent Direct Strategy (DS). The asocial condition is represented by solid lines and solid triangles; while the Social condition is represented by dashed lines and open triangles. **a,** Age trajectories for the Pin Box task. N_BaYaka_ = 45; N_Bandongo_ = 47; N_German_ = 52. **b,** Age trajectories for the Eco LS-DS task. N_BaYaka_ = 50; N_Bandongo_ = 51; N_German_ = 50. **c,** Age trajectories for the Lilypad task. N_BaYaka_ = 51; N_Bandongo_ = 51; N_German_ = 52. **d,** Age trajectories for the Maze task, N_BaYaka_ = 47; N_Bandongo_ = 50; N_German_ = 49.
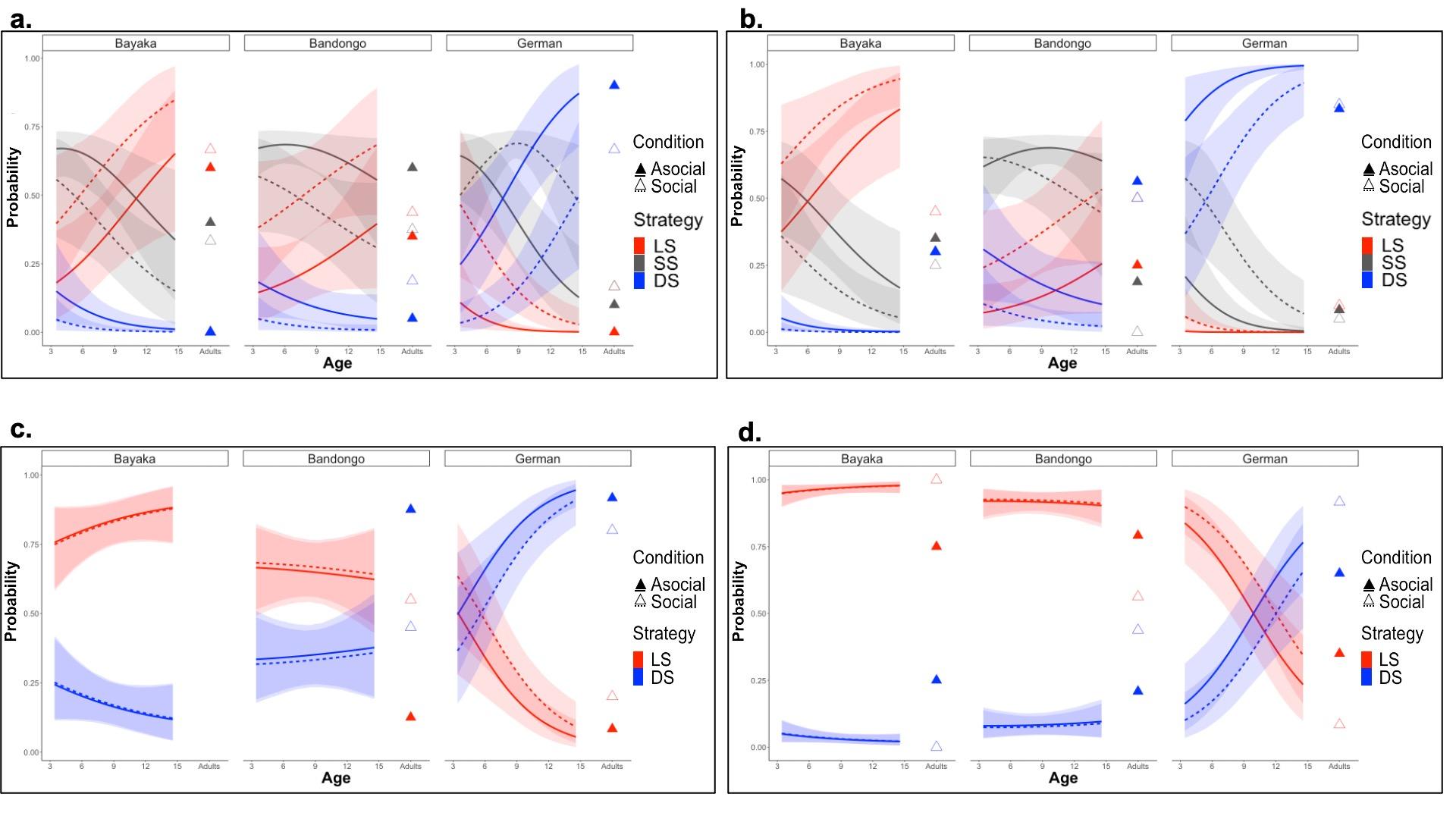


**
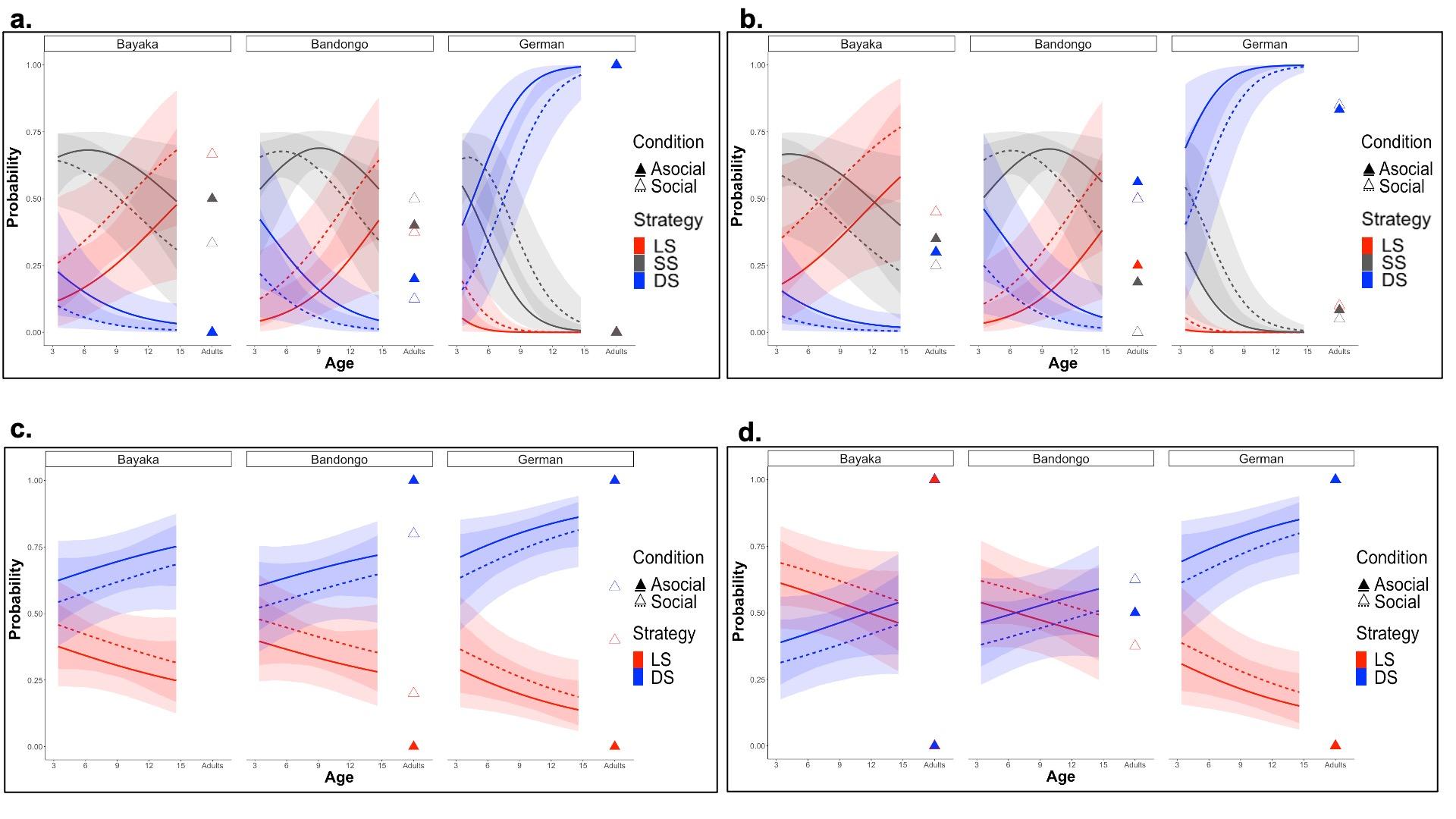
**

**Supplemental Fig.4: Elective flexibility developmental trajectories - Post-Extinction Phase: a,b,c,d,** show posterior estimates of the probabilities of using a given strategy at each given age by children of different cultural backgrounds (egalitarian BaYaka, hierarchical Bandongo, and hierarchical German). Lines show posterior means and ribbons 95% HPD intervals. The trajectories shown are for the middle  trial of the Post-Extinciton phase for each of the tasks. Red lines and triangles represent Learned Strategy (LS), grey lines and triangles represent Switch Strategy (SS) (only present for Pin Box and Eco LS-DS tasks) and blue lines and triangles represent Direct Strategy (DS). The asocial condition is represented by solid lines and solid triangles; while the social condition is represented by dashed lines and open triangles.. **a,** Age trajectories for the Pin Box task, N_BaYaka_ = 45; N_Bandongo_ = 47; N_German_ = 50. **b,** Age trajectories for the Eco LS-DS task, N_BaYaka_ = 50; N_Bandongo_ = 51; N_German_ = 50. **c,** Age trajectories for the Lilypad task, N_BaYaka_ = 51; N_Bandongo_ = 51; N_German_ = 52. **d,** Age trajectories for the Maze task, N_BaYaka_ = 46; N_Bandongo_ = 50; N_German_ = 49.

### Appendix 4

**Tasks Descriptions:**

**a) Eco LS-DS:** For this computer task, participants complete 15 trials on a touchscreen depicting three white squares and one image (palm tree), one in each quadrant. In the Acquisition phase (AP) children learn to select the palm tree image, to uncover a second image (tree); upon selecting the second image, the third image (cactus) is revealed. After selecting all three images, the location of the goal object (ball) is revealed. This four-step Learned Strategy (**LS**) is learned either by themselves (Asocial condition) or after a demonstration from the experimenter (Social condition). For the next seven trials, children must use the four-step LS to collect balls. But in trials 9-12 (Test Phase - TP), the ball is visible from the beginning of the trial. Thus, participants can either use the LS, or they can simply select the already visible ball, the one-step Direct Strategy (**DS**). In trial 13, the Extinction phase (EP), participants can only use the one-step DS to solve the task. Finally, in trials 14 and 15 (Post-Extinction phase - PEP), participants are presented again with both LS and DS, similar to the DS phase. Once the child has finished all 15 trials, the experiment ends.


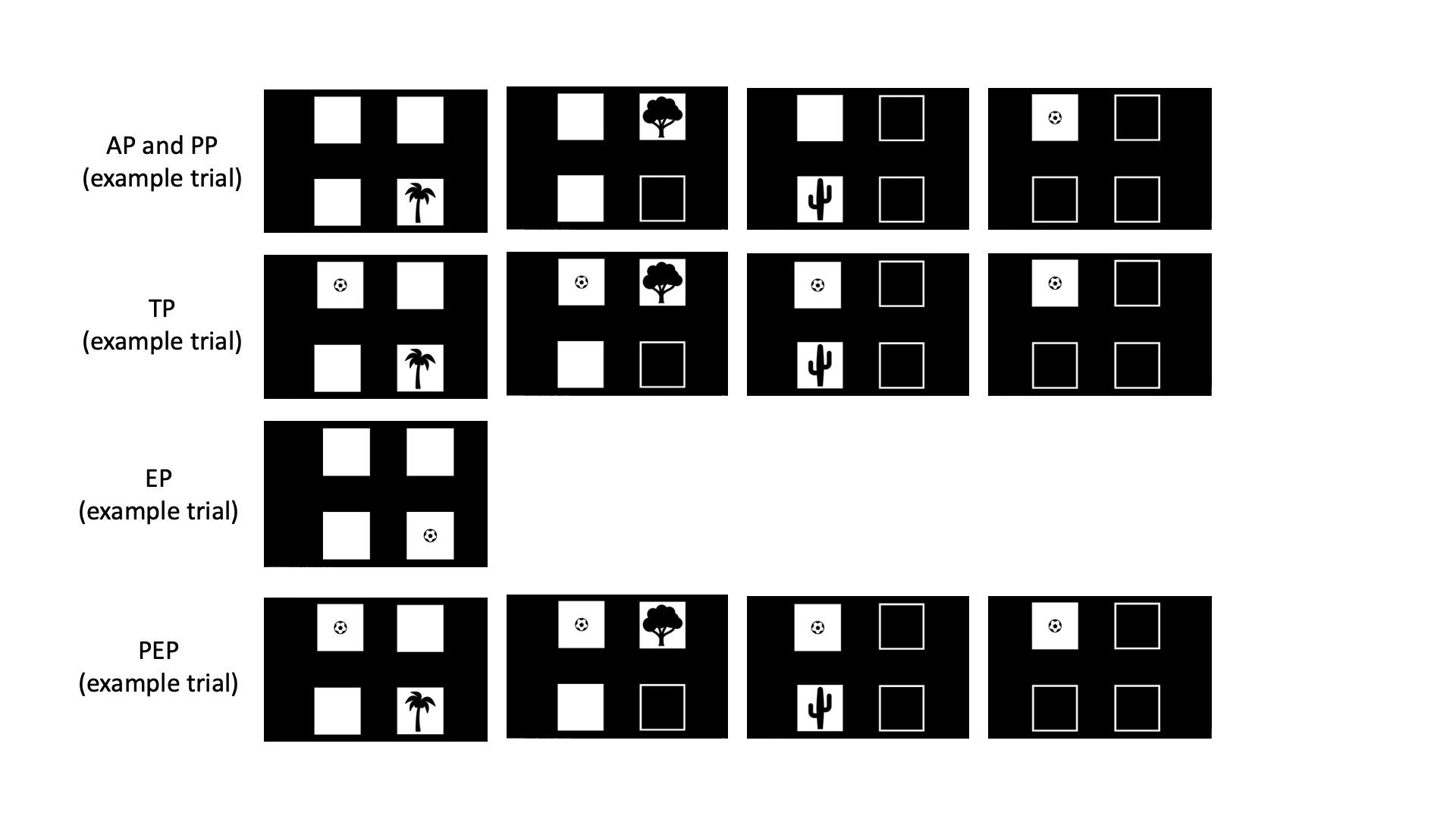


**b) Maze:** For this computer task, participants solve a set of 15 mazes presented on a touchscreen. Each maze is comprised of three trails made up of differently shaped ‘stones’ marked by unique colors. To move along the trails, participants must select an adjacent stone. In the Acquisition phase, participants learn to use the 10-step red trail to collect the goal object (ball), either by themselves (Asocial condition) or after a demonstration from the experimenter (Social condition). The first seven mazes are solved only using the 10-step red trail (LS), while the purple and blue trails were incomplete and therefore did not lead toward the ball. In mazes 9-12, the four-step blue trail (DS) can also be used to reach the goal, so participants can choose to use either the LS or the DS. Maze 13 can only be solved by using the DS. Finally, on mazes 14 and 15 (Post-Extintion phase), participants are presented again with both LS and DS. Once the child has finished all 15 trials, the experiment ends.


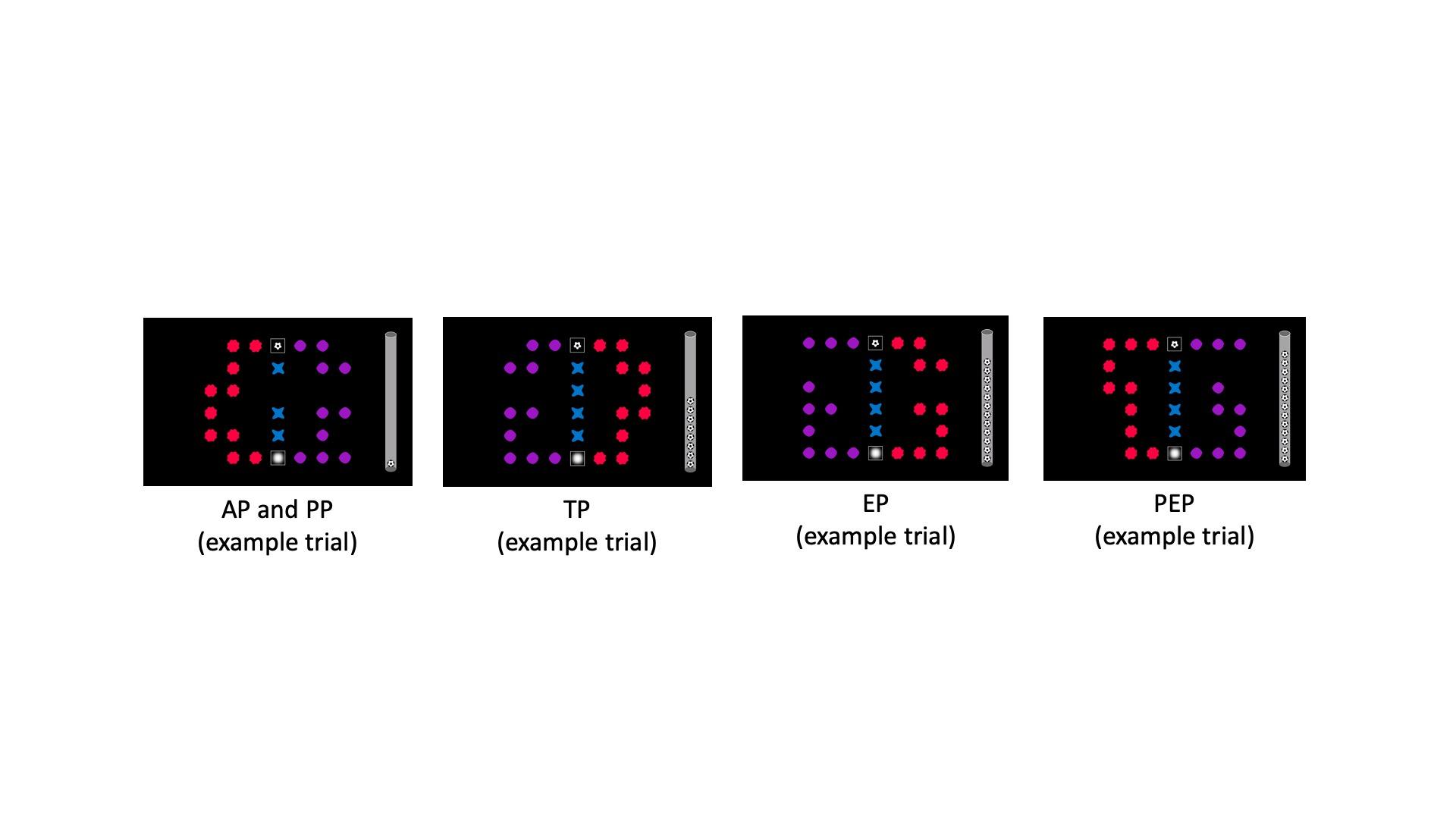


**c) Pin Box:** For this hands-on task, participants try to extract a goal object (ball) from a closed box, which is held closed by two pins and a transparent lid. In the Acquisition phase, participants learn to pull out the purple pin first, which then allows them to remove the green pin. Once both pins are removed, participants can open the transparent lid and reach into the box to collect the ball. The four-step purple-green-lid-token (LS) is learned either by themselves (Asocial condition) or after a demonstration from the experimenter (Social condition). For the next seven trials, participants can only use the LS to open the box and collect the token. From trials 9-12, the transparent lid is replaced by an empty frame in which there is no plexiglass to block access to the inside of the box. Thus, participants can either directly access the token by reaching into the box, the one-step DS, or they can use the four-step LS. On trial 13, the box is presented without the lid nor the pins, thus, only solvable by using the DS. Finally, on mazes 14 and 15 (Post-Extinction phase), participants are presented again with both LS and DS. Once the child has finished all 15 trials, the experiment ends.


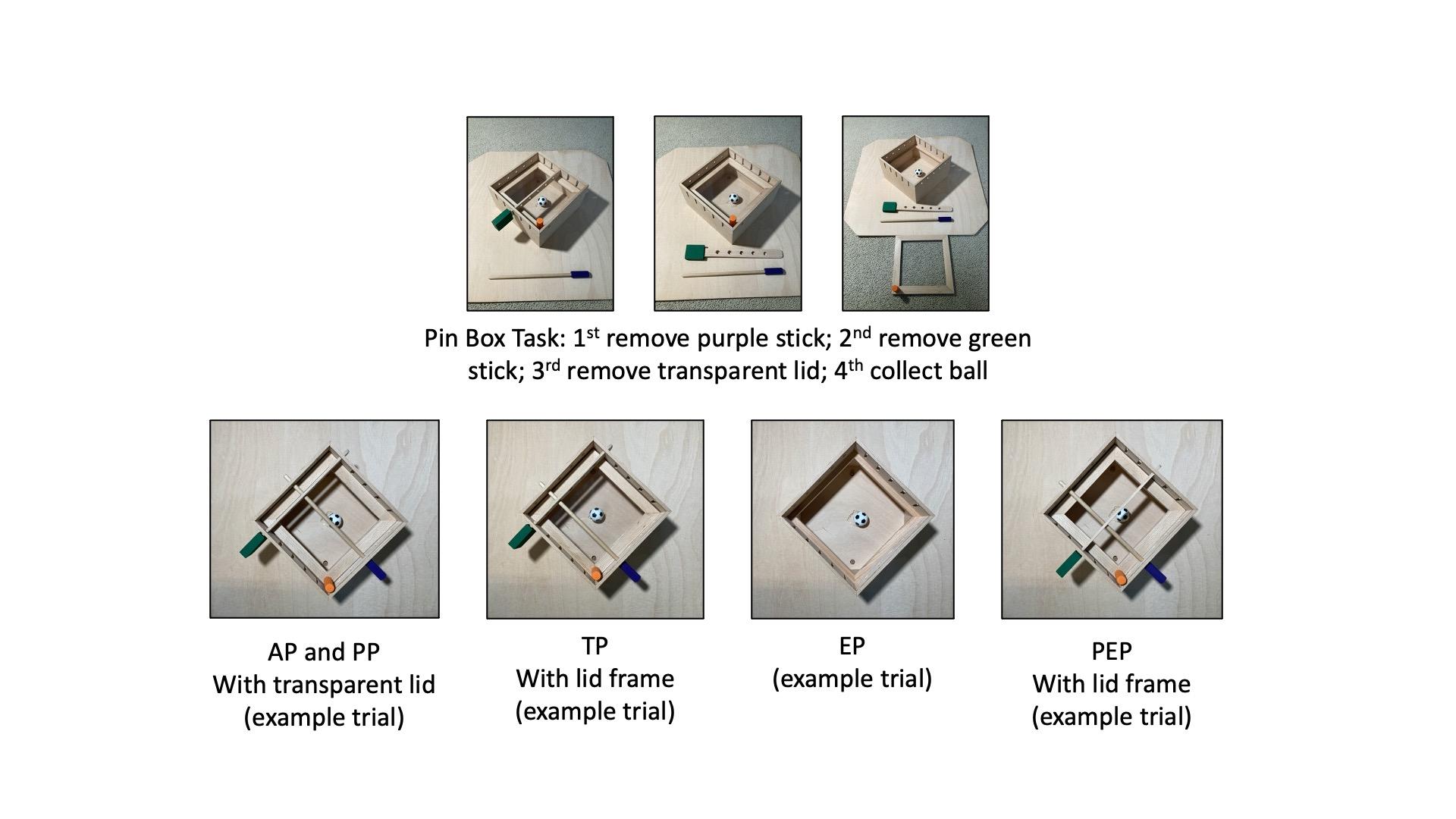


**d) Lilypad:** For this task, participants walk through 15 mazes (4x4 meters) displayed on tarps laid on the ground. Each maze consists of three paths leading from the start to the goal. To complete each trial, participants collect a ball at the other end of the maze and return with it to the start. In the Acquisition phase, participants learn to use the 8-step yellow path, either by themselves (Asocial condition) or after a demonstration from the experimenter (Social condition). During this phase, the other two paths (red and blue) are ineffective (each is missing a necessary stepping ‘stone’). At the end of each trial, a new maze is revealed by unrolling the next tarp. In the next seven trials, participants can only use the 8-step LS to solve the task. In trials 9-12, the four-step red path (DS) is available. Thus, participants can choose to either use the eight-step LS or they can use the four-step DS to collect the ball. For trial 13, participants can only use the DS to solve the task. Finally, on mazes 14 and 15 (Post-Extinction phase), participants are presented again with both LS and DS. Once the child has finished all 15 trials, the experiment ends.


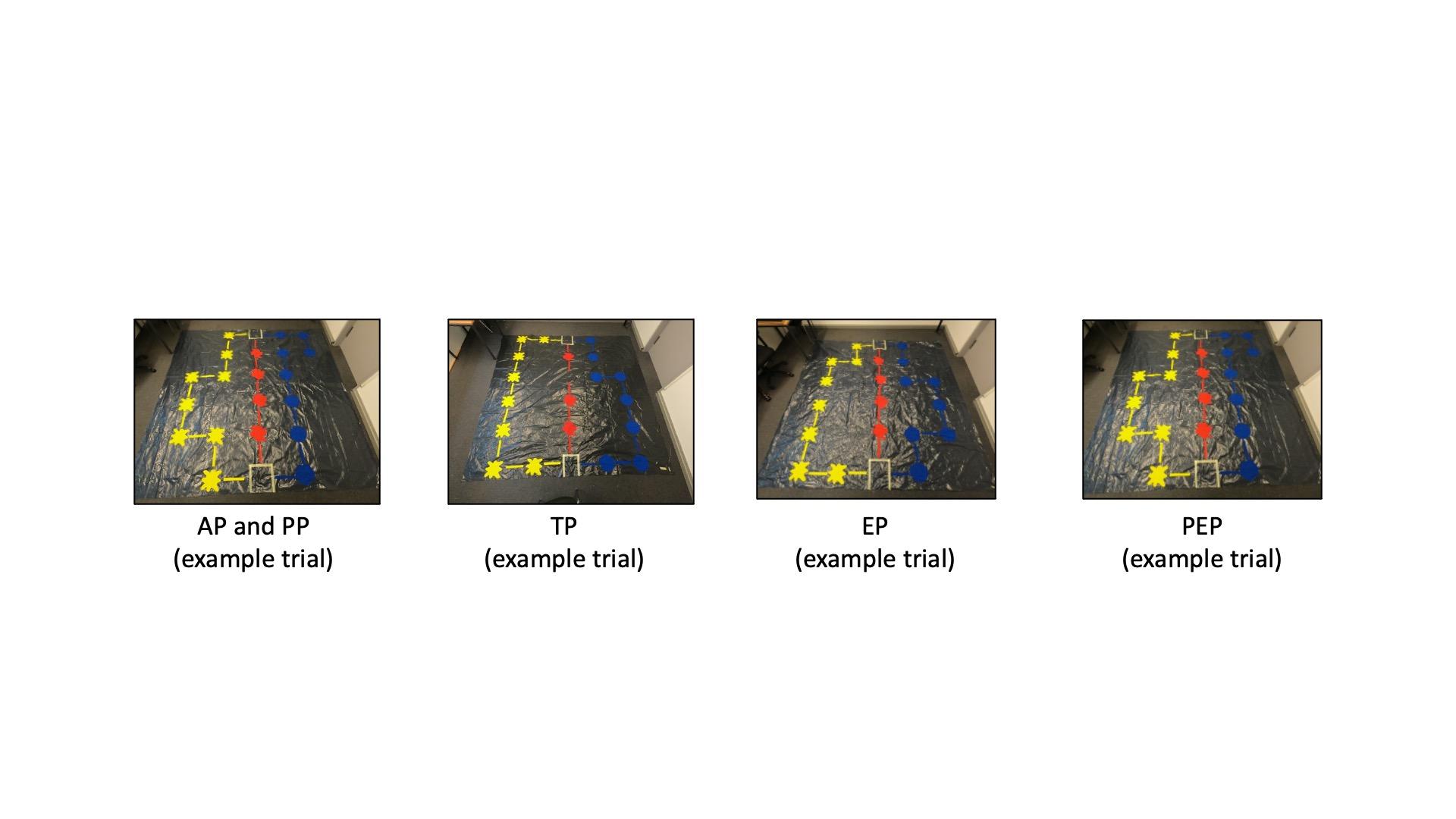

Supplement: Supplementary file 1 — Supplementary Material 1 [file 41598_2025_15400_MOESM1_ESM.docx]
